# Mindfulness-Based Ecological Momentary Intervention for Smoking Cessation to Address Cancer-Related Relapse Risk Factors: Intervention Development and Usability Findings

**DOI:** 10.1007/s12671-026-02775-0

**Published:** 2026-03-09

**Authors:** Min-Jeong Yang, Steven K. Sutton, Cherell Cottrell-Daniels, Lee M. Ritterband, Rebecca Blackwell, Melinda Leigh Maconi, Ranjita Poudel, Smitha Pabbathi, Vani N. Simmons, Christine Vinci

**Affiliations:** 1 Institute for Nicotine and Tobacco Studies, Rutgers Health, Rutgers, The State University of New Jersey, New Brunswick, NJ USA; 2 Department of Health Behavior, Society, and Policy, School of Public Health, Rutgers, The State University of New Jersey, Piscataway, NJ USA; 3 Department of Biostatistics and Bioinformatics, Moffitt Cancer Center, Tampa, FL USA; 4 Department of Oncologic Sciences, University of South Florida, Tampa, FL USA; 5 Department of Psychology, University of South Florida, Tampa, FL USA; 6 Research Department, Health Choice Network, Miami, FL USA; 7 Department of Psychiatry and Neurobehavioral Sciences, School of Medicine, University of Virginia, Charlottesville, VA USA; 8 Department of Sociology, University of South Florida, Tampa, FL USA; 9 Participant Research, Intervention, and Measurements Core, Moffitt Cancer Center, Tampa, FL USA; 10 Department of Health Outcomes and Behavior, Moffitt Cancer Center, Tampa, FL USA; 11 Internal Medicine Residency Program, St. Joseph’s Main Hospital, BayCare Health System, Tampa, FL USA

**Keywords:** Smoking Cessation, Cancer, Smartphone application, Cancer-related difficulties

## Abstract

**Objectives:**

Despite significant potential adverse health outcomes, many cancer survivors continue smoking. Few smoking cessation interventions have demonstrated efficacy above standard treatment for this population. Through a rigorous iterative process, we developed a prototype smartphone app that addresses both general and cancer-specific relapse risks in real time, incorporating cancer survivors’ feedback. We report findings from two studies: (1) Qualitative interviews and brief surveys to inform intervention development and (2) Usability testing of the prototype app in a 4-week single-arm pilot trial.

**Method:**

Cancer survivors (Study 1: *n* = 20; Study 2: *n* = 12) who had smoked at least one cigarette within the past 30 days were enrolled. Study 1 participants completed a 50-minutes Zoom interview and survey to inform intervention content and the app design. Subsequently, a prototype app was developed and tested for usability in Study 2, which included ecological momentary interventions (EMIs), three telehealth counseling sessions, and nicotine patches. Baseline and end-of-treatment (EOT) surveys and interviews were completed.

**Results:**

Key findings in Study 1 included high perceived helpfulness of mindfulness and EMIs for managing cravings and cancer-related stress, and a reported strong willingness to use the app. In Study 2, eleven participants used the app and completed counseling sessions. Participants reported high treatment acceptability, app usability, and ease of use. A key suggested improvement was adjusting the timing of app notifications.

**Conclusions:**

Results support continued investigation of this app. Next steps include testing the feasibility and acceptability of the app for smoking cessation.

**Preregistration:**

Study 2 is registered in ClinicalTrials.gov (NCT06476548).

**Supplementary Information:**

The online version contains supplementary material available at 10.1007/s12671-026-02775-0.

Continued smoking after a diagnosis of cancer is linked to a reduced effectiveness of cancer treatment, an increased risk of cancer-related mortality (Florou et al., 2014; US Department of Health & Human Services, 2020), and increased cancer treatment costs ($3.4 billion/year; Warren et al., 2019). Despite the adverse health outcomes associated with continued smoking, many cancer survivors continue smoking (Gritz et al., 2020). Among those who make a quit attempt, the relapse rate is as high as 57% (Feuer et al., 2022; U.S. National Cancer Institute, 2022), which is higher than or comparable to the rate observed in the general population (Alboksmaty et al., 2019; U.S. National Cancer Institute, 2022). Smoking cessation interventions with behavioral techniques known to be effective in other populations have not demonstrated efficacy above standard treatment in this population (Sheeran et al., 2019). Consequently, there is a need to develop effective smoking cessation interventions that address both general (e.g., cravings) and cancer-specific relapse (e.g., fear of cancer recurrence) risk factors (Andersen et al., 1994; Berg et al., 2013; Cooley et al., 2009; Feuer et al., 2022; Gritz et al., 1999; Guimond et al., 2017; Jassem, 2019; Wells et al., 2017).

Mindfulness is a promising approach given its strong evidence in decreasing emotional difficulties among cancer survivors (Carlson et al., 2023) and reducing reactivity to craving and stress among individuals who smoke (Garrison et al., 2020; Kober et al., 2017). Both formal meditations and informal mindfulness practices (e.g., briefly bringing awareness to present-moment activities such as breathing or walking) that address the automaticity of target behaviors (e.g., smoking) are key intervention components in mindfulness-based interventions (MBIs). Randomized controlled trials (RCTs) for smoking cessation incorporating key mindfulness content have shown the efficacy of MBIs in biochemically verified abstinence and relapse prevention at follow-up after completion of treatment (Brewer et al., 2011; Davis et al., 2014; Vidrine et al., 2016; Weiss de Souza et al., 2020), reduction in smoking (Brewer et al., 2011; Ruscio et al., 2016), and changes in theoretically relevant cognitive and affective constructs (e.g., cravings, mindfulness; Garrison et al., 2020; Weiss de Souza et al., 2020).

The application of MBIs to cancer survivors for smoking cessation is limited to two single-arm, small-scale feasibility studies (Charlot et al., 2019; Jackson et al., 2024). Few studies have targeted smoking vulnerabilities unique to cancer survivors, although the literature documents several unique cancer-related difficulties, such as the stress of cancer diagnosis, cessation barriers (e.g., shame), and emotional difficulties as relapse risk factors (Andersen et al., 1994; Berg et al., 2013; Cooley et al., 2009; Feuer et al., 2022; Gritz et al., 1999; Guimond et al., 2017; Jassem, 2019; Wells et al., 2017). Further, low attendance rates in intensive treatment formats (e.g., 8-week, 2-hr group sessions) coupled with minimal integration of MBI content in prior studies pose potential barriers to accessibility and limit the interpretation of findings.

Mobile technology may increase accessibility and provide MBIs in a real-world context. To date, two full-scale RCTs of mobile health (mHealth) MBIs for smoking cessation showed promising results in the general population (Black & Kirkpatrick, 2023; Garrison et al., 2020). As compared to attention control conditions, MBIs weakened the association between craving and smoking (Garrison et al., 2020) and resulted in a greater reduction of cigarettes per day (Black & Kirkpatrick, 2023), although the latter study lacked smoking-related intervention content (Black & Kirkpatrick, 2023). Only a few single-arm feasibility MBI studies have leveraged opportunities to intervene on smoking in real time by detecting high-risk moments of relapse using wearable sensors in the general population (Horvath et al., 2024; Vinci et al., 2025; Yang et al., 2023). These studies demonstrated momentary improvements in state mood (Horvath et al., 2024; Vinci et al., 2025) and cravings (Horvath et al., 2024) after engaging in the real-time MBI. Leveraging mHealth to deliver MBIs by targeting both general and cancer-specific smoking vulnerabilities in real time provides a novel approach that holds promise for increasing the likelihood of abstinence among individuals with a cancer diagnosis.

This paper presents a two-phase study that aimed to develop an adjunctive mindfulness-based ecological momentary intervention (EMI) for smoking cessation among cancer survivors – defined as individuals who have been diagnosed with cancer (U.S. National Cancer Institute, n.d.). In Study 1, we interviewed and surveyed cancer survivors to gather feedback on the planned intervention content and smartphone app we designed. This input informed the development of a prototype app. In Study 2, we conducted a single-arm usability trial. The EMI was delivered via a smartphone app in combination with brief counseling and nicotine replacement therapy, which are considered standard care (Shields et al., 2023). Our approach aligned with the Accelerated Creation-to-Sustainment (ACTS) model’s Create phase in digital intervention development, which focuses on developing a minimally viable technology-enabled intervention and identifying initial implementation strategies (i.e., engagement; Mohr et al., 2017).

## Study 1

### Method

#### Participants

Inclusion criteria were (1) being ≥ 18 years old, (2) having smoked at least one cigarette (even one or two puffs) in the past 30 days per the guidelines in the literature (NCI–AACR Cancer Patient Tobacco Use Assessment Task Force, 2016; Shields et al., 2023), (3) having been diagnosed with cancer, (4) having a valid home address and functioning phone number, (5) being able to read, write, and speak English, and (6) having a smartphone. The exclusion criterion was current enrollment in a smoking cessation program. This study was approved by the Advarra Institutional Review Board (Pro00068138).

#### Procedure

Potential participants were identified via medical record review. Once deemed preliminarily eligible (i.e., age, diagnosis, current smoking status), they were contacted via phone to learn about the study, determine eligibility, and, if eligible, to obtain verbal informed consent. Consented participants were invited to complete an individual interview conducted over Zoom and to complete a brief REDCap survey (~ 10 minutes). Participants were provided a $25 digital debit card for completing both the interview and survey, with an additional $5 if the survey was completed within 24 hr after the survey link was sent.

Informed by the Technology Acceptance Model (Davis, 1985), a single semi-structured interview assessed the perceived usefulness of planned MBI content to quit smoking, the perceived ease of use and usefulness of a planned smartphone app to practice mindfulness and quit smoking, and preferences on the smoking cessation counseling modality (phone vs. virtual). During the interview, participants were presented with app wireframes and engaged in a brief formal meditation (5-minutes guided body scan meditation) and practiced a set of two mindful skills from each of four different categories: three cancer-related topics (fear of cancer recurrence, pain, and fatigue) and one self-compassion topic. Timing and delivery of EMIs—mindful skills delivered in text format—and ecological momentary assessments (EMAs) were also presented to capture moments of heightened vulnerabilities, with an EMI delivered in response.

#### Measures

##### Clinical Characteristics

Clinical variables were obtained via chart abstraction completed via clinical data abstractors and included the most recent primary cancer site, diagnosis date, and disease stage using the Tumor–Node–Metastasis (TNM) staging system.

##### Brief Survey

Self-reported demographic information was obtained (e.g., date of birth, gender identity, race, ethnicity). Previous experience with mindfulness practices was measured using items from our previous study (Vinci et al., 2020). For smoking characteristics, the Heaviness of Smoking Index (Borland et al., 2010; Heatherton et al., 1989; Cronbach’s α = 0.32 in the current sample) was used to assess nicotine dependence; the Contemplation Ladder (Biener & Abrams, 1991) was used to assess readiness to quit smoking; and the Cancer Patient Tobacco Use Questionnaire (C-TUQ; Land et al., 2016; NCI-AACR Cancer Patient Tobacco Use Assessment Task Force, 2016) was used to measure tobacco use among cancer patients. To measure the perceived usefulness of the planned smartphone app, a team-developed item (“How likely would you be willing to try an app like the one we described to learn how to use mindfulness to quit smoking?”) assessed willingness to use the app (1 = *not very likely*, 6 = *very likely*). Three items, modified from a perceived usefulness subscale of the validated Technology Acceptance Model Questionnaire (Davis, 1989) assessed the perceived usefulness of the app (1 = *strongly disagree*, 7 = *strongly agree*).

#### Data Analyses

Participant characteristics and quantitative data were analyzed to derive descriptive summary statistics. All semi-structured interviews were audio-recorded, transcribed verbatim, and de-identified. NVivo 12 (Lumivero, 2025) was used for qualitative data analysis following guidelines on applied thematic content analysis (Guest et al., 2011; Strauss & Corbin, 1998). Two coders (the first and third authors) independently reviewed transcripts and generated initial codes. They collaboratively developed a codebook (containing a priori and emergent codes), iteratively refined across five transcripts, and achieved high interrater reliability (*Kappa* > 0.80; McHugh, 2012). Following the consensus, the remaining transcripts were then divided for independent coding.

### Results

#### Participant Characteristics

Of the 123 patients deemed preliminarily eligible, 60 completed the phone screen, among whom 32 were eligible, 24 consented (*n* = 8 not interested), and 20 completed both the interview (41–80 minutes) and the online survey. On average, participants reported smoking 13.50 (*SD* = 7.97) cigarettes per day when they smoke. The majority of participants (75.00%) reported smoking during their cancer treatment. Table 1 presents the demographic, clinical, and smoking characteristics of the sample.

**Table 1:** Study 1: Demographic Characteristics (*n* = 20)

Variables	*M* (*SD*) or *n* (%)
**Age**	63.70 (8.90)
**Hispanic/Latino**	1 (5.00%)
**Race**	
Native Hawaiian or Other Pacific Islander	1 (5.00%)
White	19 (95.00%)
**Sex assigned at birth** (Female)	11 (55.00%)
**Gender identity** (Female)	11 (55.00%)
**Sexual orientation** (heterosexual)	20 (100.00%)
**Education** (≤ High school/GED)	12 (60.00%)
**Household income** (< $40,000)^a^	6 (30.00%)
**Smartphone type** (Android)	11 (55.00%)
**Mindfulness meditation**^b^	
Never heard of this	7 (35.00%)
Have heard of this but never done	10 (50.00%)
Currently do this practice	2 (10.00%)
**Clinical Variables**	
**Cancer type**	
Lung	6 (30.00%)
Breast	4 (20.00%)
Colon	3 (15.00%)
Gynecological	2 (10.00%)
Skin	2 (10.00%)
Bladder	1 (5.00%)
Kidney	1 (5.00%)
Rectum	1 (5.00%)
**Time since diagnose** (≤ 1 year)	13 (65.00%)
**Cancer stage**^c^	
0	1 (5.00%)
1	6 (30.00%)
2	3 (15.00%)
3	2 (10.00%)
4	4 (20.00%)
**Smoking Variables**	
**Heaviness of smoking index**	1.95 (1.32)
**Current average number of cigarettes per day when they smoke**	13.50 (7.97)
**Contemplation ladder**	6.05 (2.98)
**Number of years smoked cigarettes**	39.30 (12.36)
**Smoked every day or some days around the time of the most recent cancer diagnosis**^**d**^	
The year before you were first told you had cancer	20 (100.00%)
After diagnosis and before treatment started	20 (100.00%)
During the course of treatment	15 (75.00%)
After treatment ended	17 (85.00%)
**The longest time stayed off cigarettes since first learned that you had cancer (the most recently diagnosed cancer)**	
< 1 day	7 (35.00%)
1–7 days	5 (25.00%)
< 1 month	2 (10.00%)
< 1 year	2 (10.00%)
More than 1 year	4 (20.00%)
**Perceived Usefulness of App**	
**Willingness to try the app** (≥ 4 out of 6)	16 (80.00%)
**App would help me to practice mindfulness daily** (≥ 5 out of 7)	15 (75.00%)
**App would increase chance to practice mindfulness** (≥ 5 out of 7)	15 (75.00%)
**I would find this type of app useful** (≥ 5 out of 7)	15 (75.00%)

Note

^a^*n* = 5 preferred not to answer

^b^*n* = 1 missing

^c^*n* = 4 were diagnosed elsewhere, thus information obtained was via medical chart review, and no stage data was available

^d^The two following choices, i.e., “smoked every day” and “smoked some days” were combined.

#### Qualitative Results

Four major themes were identified regarding the perceived helpfulness of mindfulness and app use for smoking cessation: (1) perceived usefulness of mindfulness for smoking cessation, (2) acceptance of mHealth, (3) quitting challenges, and (4) suggestions for app and intervention content. Representative quotes are presented in Online Resource 1.

##### Theme 1. Perceived usefulness of mindfulness for quitting smoking for cancer survivors

 Most participants reported being unfamiliar with mindfulness, although they associated mindfulness with positive connotations (e.g., calm, relaxation); few noted negative reactions. In response to the meditation and mindful skills, most had a positive response (e.g., increased awareness of the body; potential helpfulness in breaking out of automatic behavior). Many found the meditation and skills easy to understand and expressed interest in practicing them. Although participants reported that mindfulness could help in stress management and for quitting smoking (e.g., pausing before engaging in automatic smoking; staying with craving to allow it to dissipate), responses were mixed regarding its ability to address cancer-related difficulties. Topics that mindfulness could address included fear of cancer recurrence, pain, and fatigue, whereas using a mindfulness-based approach for addressing shame and guilt related to smoking was somewhat less desired.

##### Theme 2. Acceptance of mHealth

Participants indicated that the EMI would be useful to pause and pay attention to present-moment experiences, although some said the timing of the EMI delivery (e.g., driving) or the intensity of pain may interfere with EMI engagement. A few mentioned that the reminders from an app would encourage them to practice mindfulness. Participants found the proposed app easy to use, user-friendly, simple, and novel. Many said EMAs would be feasible and were interested in trying the proposed app. However, a few found the app design (EMA, EMI schedules) too busy and felt that the cancer-related content did not relate to their smoking experience. A few said they are not “an app person,” thus were not interested.

##### Theme 3. Quitting challenges

Many participants mentioned challenges in quitting smoking. Some shared past quit attempts due to cancer-related surgery, cancer diagnosis, and the health of loved ones, although smoking relapse was common (e.g., reactions to cancer recurrence). A few were not interested in quitting because of having terminal cancer or perceiving their cancer as irrelevant to their smoking. Most mentioned general smoking vulnerabilities such as habitual and automatic smoking, cravings triggered by factors such as time of day, places (e.g., car), negative affect (e.g., stress, boredom), and anticipation that smoking would make them feel better. Regarding cancer-related smoking vulnerabilities, some participants did not see cancer-related difficulties as contributing to their current smoking behaviors when explicitly asked. However, many endorsed experiencing fear of cancer recurrence, pain, and fatigue, which they felt might be relevant to their smoking. In particular, several participants reported experiencing shame and guilt due to their continued smoking despite having a cancer diagnosis. Some participants mentioned that they hid their smoking from their loved ones for this reason.

##### Theme 4. Suggestions for app and intervention content

Participants preferred mindfulness practices that addressed fear of cancer recurrence, difficult emotions, and cravings, as well as being offered various lengths of meditation on the app (e.g., 5–10 minutes, 10–15 minutes). Psychoeducation on smoking cessation (e.g., triggers, pharmacotherapy), on-demand mindfulness content, and inclusion of motivational messages were suggested. A few expressed an interest in reducing the planned number of EMA items and frequency (4 times/day). Many noted the usefulness of reminder notifications to practice mindfulness. Some raised concerns about the tobacco-related language used in the study logo given the stigma and potential breaches of security. Most participants liked the idea of having counseling sessions, while half preferred phone calls, and the other half preferred Zoom for the counseling modality. Finally, participants offered suggestions for future patients, including ensuring the protection of privacy, clearly explaining the time commitment, how to manage missed app notifications, encouraging constant progress, sharing scientific evidence on quitting and health improvements, and offering detailed instructions for users with low digital literacy.

#### Brief Survey Results on Perceived Usefulness of Planned Smartphone App

More than two-thirds of participants reported that they were willing to try the app to quit smoking and believed that using the app would help them practice mindfulness daily and increase their chances of practicing mindfulness (Table 1). Many found this planned app useful.

### Discussion

The results of the qualitative interviews in Study 1 showed that both the mindfulness approach and mHealth for quitting smoking were generally perceived as acceptable and novel approaches. Despite questioning the association between cancer-related difficulties and current smoking, fear of cancer recurrence, pain, fatigue, and shame were endorsed as potential cessation barriers and relapse risks. This was corroborated by the high endorsement rate of continued smoking during cancer treatment in the brief survey findings. Prior research has demonstrated that emotional difficulties associated with cancer diagnosis and treatment may contribute to continued smoking and relapse (Berg et al., 2013; Wells et al., 2017). The observed high relapse rate among cancer survivors underscores the importance of continued support for quitting, addressing this population’s unique concerns (Feuer et al., 2022). Mindfulness delivered in real time, addressing both risk factors, was perceived to interfere with the automaticity of smoking behavior, potentially facilitating quitting. Together, these results and participant feedback guided the development of the intervention, as outlined below.

#### Overview of Feedback Considered in Intervention Development

A prototype app was developed incorporating the suggestions from the qualitative interviews. Specifically, within the app, we ensured there were no cues about smoking in the study logo, streamlined the app registration process to accommodate various levels of digital literacy, collected no identifiable participant data in the app, provided a brief app overview video to facilitate navigating the app and setting up the expectations for time commitment, included motivational messages in the EMI, provided guided meditations in both 5–6 and 10–15 minutes lengths, and made the intervention content available on-demand. During the counseling sessions, we provided psychoeducation on the patch use and coping with cravings. Three 30-minutes counseling sessions were provided in accordance with the number of weeks EMIs were delivered. We additionally provided psychoeducation on the following topics if participants brought them up during the counseling sessions: common cancer-related difficulties, benefits of quitting, and what to do if unable to respond to the app’s notifications. The counseling modality remained flexible (phone or Zoom) to accommodate participants’ preferences and schedules.

#### Mindfulness Intervention Content

The intervention content within the app was developed based on our previous work (Yang et al., 2022, 2023) and extant mindfulness-based literature (Bowen et al., 2021; Carlson & Speca, 2011; Kabat-Zinn, 2013, 2023; Lehrhaupt & Meibert, 2017; Linehan, 2014; Roos et al., n.d.), considering Study 1 feedback. Overall, there were two types of intervention content: (1) Mindful skills and motivational messages in the form of text and (2) Formal meditations in the form of audio recordings. The mindful skills included content from 14 topic areas that included 193 messages in total. There were 32 motivational messages. All meditations from 11 topic areas were available in both short (5–6 minutes) and long (10–15 minutes) lengths. Over time, different types of meditation became available. Online Resource 2 presents the full intervention content and related details.

#### Prototype App Development

Metricwire smartphone app, Catalyst (Metricwire Inc., n.d.), was used to develop the prototype app. This app platform supports both Android and Apple operating systems. Online Resource 3 shows the sample app screenshots and the EMI flow with EMA items.

#### Mindful Skills within Prototype App

Mindful skills were delivered in two ways for 3 weeks: EMI and randomly. We chose a 3-week delivery period based on our previous work (Yang et al., 2023) and early just-in-time adaptive intervention studies for smoking cessation among the general population (Businelle et al., 2016; Hébert et al., 2020). An EMI was pushed when EMA responses were ≥ 4 on an 11-point Likert scale (0 = *not at all* to 10 = *Extremely*) in response to one of 8 EMA items. The 8 EMA items addressed three target constructs: cancer-related concerns, craving, and negative affect. When a participant reported ≥ 4, a single mindful skill was randomly drawn from the assigned topic areas and pushed. To ensure that the mindful skill corresponded with the EMA response, the mindful skill topic area was matched to the EMA response (e.g., if a 5 was reported for craving, participants were sent a craving-related mindful skill; see Online Resource 2 for details). EMAs were pushed 4 times a day, so participants could receive up to 4 EMIs per day. Random mindful skills were pushed 2 times per day. The mindful skills were also available on demand.

#### Meditations within Prototype App

A once daily (12 pm) reminder notification to practice 10–15 minutes of meditation was sent for 4 weeks. Upon clicking the notification, participants could select a meditation to practice. Each week, new meditations were unlocked, and all unlocked meditations (both in 5–6 and 10–15 minutes durations) remained accessible on demand.

## Study 2

### Method

This study examined the usability of the app in a single-arm 4-week trial among cancer survivors. The intervention included app-based mindfulness content, three brief telehealth cessation counseling sessions, and nicotine replacement therapy. The primary outcomes were (1) retention rate as indexed by the number of participants who completed the end-of-treatment (EOT) survey, (2) perceived utility and likability of mindfulness practices, (3) participants’ feedback on the study app, and (4) open-ended feedback. We also report app engagement results and changes in smoking behavior.

#### Participants

The same recruitment method of Study 1 was used. The sample size of 13 was determined, accounting for 30% drop out, based on the recommended sample size for usability studies being at least *n* = 10 to capture 95% of potential usability problems (Faulkner, 2003). Eligibility criteria mirrored those from Study 1, with the following additional criteria: (1) Willing to make a quit attempt; (2) Having a smartphone that can download the app; (3) Willing to download and use the app daily; (4) No current use of smoking cessation medications; (5) No evidence of current psychosis; and (6) No current or planned pregnancy or lactation. Recruitment was from June 2024 to August 2024. The study was approved by the Advarra Institutional Review Board (Pro00068138) and registered in ClinicalTrials.gov (NCT06476548).

#### Procedure

**Fig. 1:**
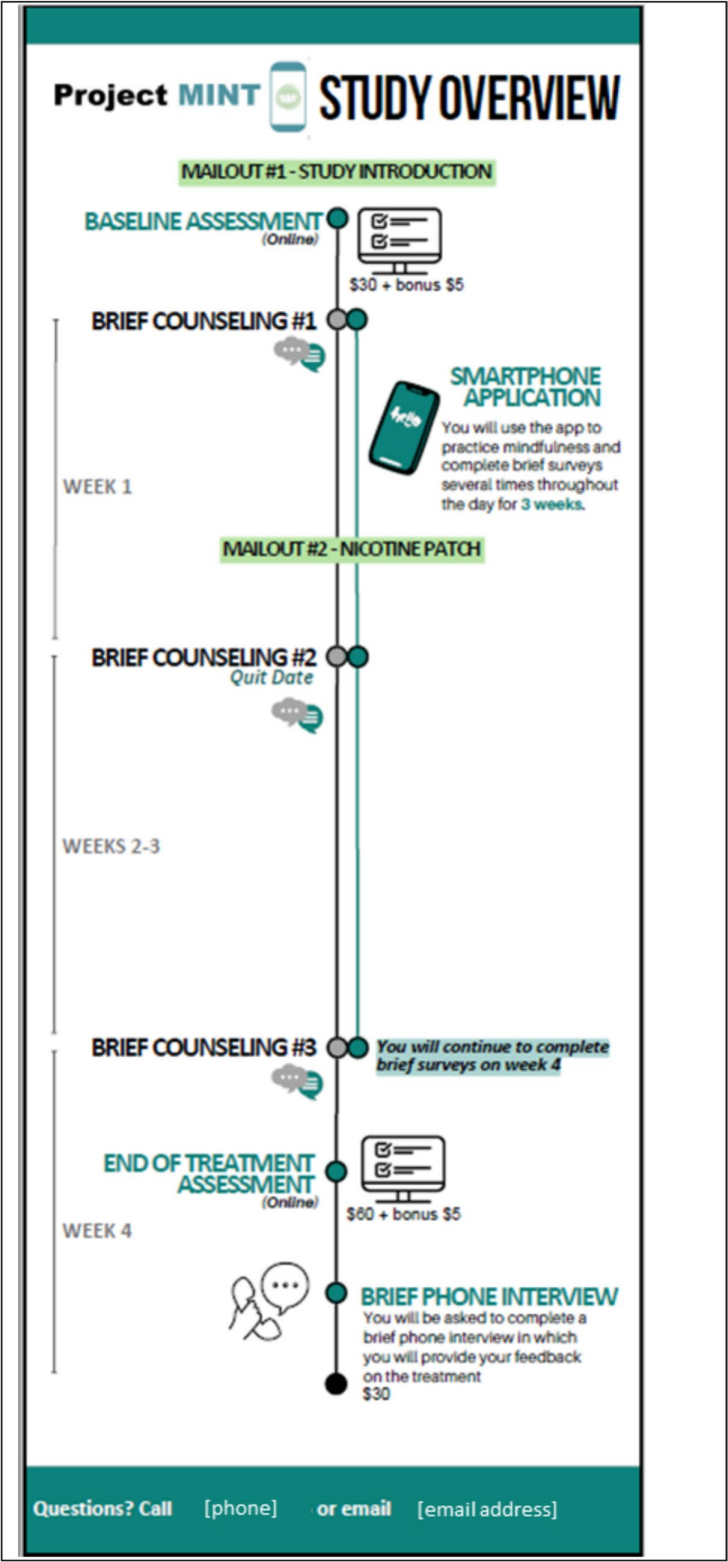
Study 2: participant flow

Figure 1 shows the overview of the participant flow through the study with compensation milestones. Eligible participants provided verbal consent, scheduled their telehealth counseling sessions (preferably via Zoom, with flexibility to use either phone or Zoom), and received a link to the baseline survey via both email and text. Quit date (QD) was scheduled 1 week after the 1st counseling session. Two mail-outs were scheduled: (1) The 1st mail-out, delivered before the 1st counseling session, contained a handout with the study overview, counseling schedule, a brief handout including psychoeducation on mindfulness, and a reloadable debit card for compensation; (2) The 2nd mail-out, delivered before the 2nd counseling session (QD), contained a 4-week supply of nicotine patches and patch instructions, and a copy of the National Cancer Institute’s Clearing the Air booklet (National Cancer Institute, 2008). The EOT survey and interview were completed during Week 4. Participants were compensated up to $130, including a $5 bonus for each survey completed within 24 hr after receiving a survey link. No compensation was provided for app use or completed EMAs.

##### Counseling & App Download

Participants attended three virtual counseling sessions led by a doctoral-level licensed clinical psychologist over Zoom or phone. Each session consisted of brief psychoeducation on smoking cessation and mindfulness, a formal meditation practice, and an inquiry process to reinforce awareness of thoughts, emotions, and physical sensations experienced during the meditation and application of mindfulness to smoking behavior (Crane et al., 2017). At the end of the 1st session, participants downloaded and installed the study app on their phone. Once the app was set up, participants were asked to play the app introduction video to confirm that the app worked correctly on their device.

##### Prototype App Use

Participants were instructed to use the app daily. Mindful skills and meditations were provided in two ways: (1) via notifications and (2) on-demand. Notification-based content included EMIs sent in response to EMA responses with ≥ 4, random mindful skills sent twice daily, and a daily meditation practice reminder. On-demand content included mindful skills and meditation practice. To encourage app use, we implemented multiple reminder strategies. If a participant had not initiated app engagement on the day of app download, a text reminder was sent the following day. If a participant did not engage with the app for 2 consecutive days, daily text reminders were sent for up to 2 additional days or until they re-engaged. If the participant still did not engage the next day, a phone call was made.

##### Nicotine Replacement Therapy

A 4-week supply of the nicotine patch was provided according to participants’ baseline cigarettes per day (CPD) level (if smoking ≤ 10 CPD, 14 mg and if smoking > 10 CPD, 21 mg).

#### Measures

##### Baseline Survey

Measures reported include demographic information, Cancer Patient Tobacco Use Questionnaire (C-TUQ; Land et al., 2016), Heaviness of Smoking Index (Heatherton et al., 1989; α = 0.36 in the current sample), a team-developed questionnaire that assessed prior experience with mindfulness meditation (Yang et al., 2023), Patient Health Questionnaire-8 (PHQ-8; Kroenke et al., 2009; α = 0.79 in the current sample) to assess depression severity, Cancer Worry Scale (CWS; Custers et al., 2018; α = 0.95 in the current sample) to assess fear of cancer recurrence, Brief Fatigue Inventory (BFI; Mendoza et al., 1999; α = 0.88 in the current sample) to assess fatigue severity, and Brief Pain Inventory (BPI; Cleeland, 1989; α = 0.90 in the current sample) to assess pain severity.

##### Ecological Momentary Assessment (EMA) within the App

Random EMAs assessed state mindfulness, affect, cravings, and cancer-related difficulties (see Online Resource 2 for details) on an 11-point Likert scale (0 = *not at all* to 10 = *Extremely*), as well as nicotine patch use (yes/no) and CPD (entered number). A total of 13 items were pushed 4 times per day, whereas the two items on nicotine patch use (starting Week 2) and CPD were pushed for the 1st EMA of the day. The follow-up EMA to an EMI contained 4 items that measured craving, distress, and the perceived timing and helpfulness of the received mindful skill, the latter two of which were rated on a 10-point Likert scale (1 = *poor timing/not at all* to 10 = *excellent timing/extremely*).

##### Inquiry Items Following Meditation Practice within the App

Following the completion of each meditation, participants answered a question randomly drawn from a pool of 2 inquiry items: What did you notice during this practice? How might this practice relate to changing smoking behaviors?

##### End of Treatment Survey

The Client Satisfaction Questionnaire (CSQ; Larsen et al., 1979; α = 0.92 in the current sample) assessed overall treatment satisfaction; the System Usability Scale (SUS; Lewis & Sauro, 2009; α = 0.96 in the current sample) and 9 items selected from the 12-item Technology Acceptance Model (TAM) questionnaire (Davis, 1989) assessed app usability, and team-developed items measured the perceived utility and likability of mindfulness practice, rated on a 6-point Likert scale (1 = *extremely unhelpful/dislike* to 6 = *extremely helpful/like*), as well as feedback on the intervention content, EMAs, and counseling sessions. As an exploratory measure, we assessed 7-day point prevalence abstinence (PPA) using two self-reported items (i.e., Number of days smoked in the past week, Average CPD in the past week on the days smoked).

##### End of Treatment Interview

A brief semi-structured interview obtained open-ended feedback on the app’s usability and overall treatment experience. All interviews were audio-recorded.

##### Clinical Characteristics

Clinical variables (the most recent primary cancer site, diagnosis date, TNM disease stage, and treatment history) were obtained using the same method as in Study 1.

#### Data Analyses

Descriptive analyses (e.g., *mean*, *median*, *SD*s) were conducted on study variables, including baseline, EOT surveys, and app engagement (e.g., EMA completion rate, random mindful skill completion rate). Medians and ranges are presented for both baseline and EOT survey results to more accurately reflect the distribution of the data. Audio-recorded interviews were transcribed verbatim and de-identified. The resulting transcripts were then analyzed using rapid analysis methods, which involve summarizing key content directly into structured templates or matrices rather than line-by-line coding to expedite interpretation while maintaining rigor (Gale et al., 2019). Focusing on participants’ perceptions and experiences related to the app, including usability, content, and timing preferences, the doctoral-level qualitative analysts (MM & RB) independently reviewed transcripts to summarize participant feedback using a standardized matrix for coding in Microsoft Excel that corresponded to interview topics. The analysts identified exemplar quotes and highlighted actionable feedback. Consensus was achieved through recurring analysis meetings and alternating transcripts in several rounds of review.

### Results

#### Participant Characteristics

Table 2 presents demographic, clinical, and smoking characteristics among the 12 patients who were enrolled (i.e., completed the baseline survey). Over 90.00% reported having smoked after diagnosis and approximately two-thirds reported having smoked during and/or after their treatment.

**Table 2:** Study 2: Demographic Information at Baseline (*n* = 12)

Variables	*Median* (*range*) or *n* (%)
**Age**	59 (42–68)
**Non-Hispanic/Latino**	12 (100.00%)
**Race**	
Black or African American	2 (16.67%)
White	10 (83.33%)
**Sex assigned at birth** (Female)	8 (66.67%)
**Gender identity** (Female)	8 (66.67%)
**Sexual orientation** (Heterosexual)	12 (100.00%)
**Education** (≤ High school/GED)	5 (41.67%)
**Household income** (< $40,000)	6 (50.00%)
**Smartphone type** (Android)	10 (83.33%)
**Number of smartphone apps used daily** (≥ 3 apps)	9 (75.00%)
**Mindfulness meditation**	
Never heard of this	2 (16.67%)
Have heard of this but never done	7 (58.33%)
Did in the past, but not currently	2 (16.67%)
Currently do this practice	1 (8.33%)
**Clinical Variables**	
**Cancer type**	
Lung	2 (16.67%)
Breast	3 (25.00%)
Gynecological	3 (25.00%)
Skin	2 (16.67%)
Bladder	1 (8.33%)
Thyroid	1 (8.33%)
**Time since diagnose** (≤ 1 year)	5 (41.67%)
**Cancer stage**^a^	
0	1 (8.33%)
1	4 (33.33%)
2	2 (16.67%)
3	1 (8.33%)
4	2 (16.67%)
Not available/unknown^a^	2 (16.67%)
**Treatment type**^**b**^	
Surgery	8 (66.67%)
Chemotherapy	6 (50.00%)
Radiation Therapy	5 (41.67%)
Immunotherapy	2 (16.67%)
Other (experimental drug)	1 (8.33%)
Unknown	1 (8.33%)
**Psychosocial Variables**	
Patient Health Questionnaire-8 Score	4.50 (0–13)
Cancer Worry Scale Score	16.50 (7–24)
Brief Pain Inventory: Pain Severity	4.25 (1.50–6.75)
Brief Fatigue Inventory: Fatigue Severity	4.44 (2.78–7.56)
**Smoking History**	
**Heaviness of smoking index**	1.50 (0–3)
**Number of days smoked in the past week**	7 (3–7)
< 7 days	3 (25.00%)
7 days	9 (75.00%)
**Number of cigarettes when smoked in the past week**	5.00 (1–20)
**Contemplation ladder**	7.50 (2–10)
**Number of total years smoked cigarettes**	30.50 (20–45)
**Smoked around the time of the most recent cancer diagnosis** (smoked every day/some days)	
After diagnosis, and before treatment started	11 (91.67%)
Two days before and after last cancer surgery	7 (58.33%)
During the course of treatment^c^	8 (66.67%)
After treatment ended^c^	7 (58.33%)
**Products used to quit smoking cigarettes since first told you had cancer (select all that apply)**	
Nicotine patch	3 (25.00%)
Nicotine gum/lozenge	2 (16.67%)
E-cigarettes or other electronic nicotine delivery system	1 (8.33%)
None	7 (58.33%)
**Have been advised to quit by healthcare providers**	10 (83.33%)
**Have been trying to quit in the past 30 days**	6 (50.00%)

^a^Individuals who were diagnosed elsewhere, thus, information is unavailable.

^b^Numbers are not mutually exclusive.

^c^Some participants reported having not received treatment (*n* = 2) or having not completed treatment (*n* = 4) for the most recent cancer diagnosis.

#### Primary Outcomes

##### Retention

**Fig. 2:**
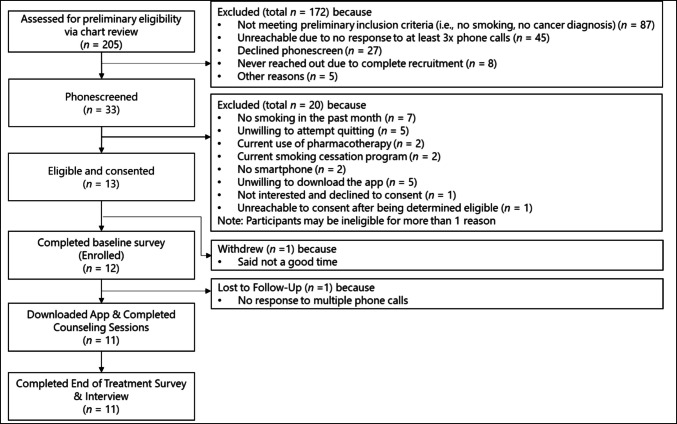
Study 2: CONSORT diagram

Figure 2 displays the CONSORT diagram for Study 2. Among 33 patients screened, 13 consented, with 12 completing the baseline survey. Among the 11 patients who downloaded the app and attended the 1st counseling session, all (100.00%) attended at least 2 counseling sessions, with 9 patients (81.82%) completing all 3 sessions, and all patients (100.00%) completed both the EOT survey and interview. Regarding the counseling modality, the majority used Zoom, and two participants consistently used the phone for all sessions.

##### Perceived utility and likability of mindfulness practice

Table 3 shows the overall results. The CSQ indicated high treatment satisfaction. The overall perceived helpfulness and likability of mindfulness practice were high. Participants found mindfulness practices helpful for decreasing smoking behavior. As shown in Online Resource 4, most participants found that the number of brief mindful skills each day and the variety in content were just right, although the responses to the timing of the delivery of the mindful skills were somewhat mixed. Regarding meditations, the majority found that the variety in content and length was just right.

**Table 3:** Study 2: End of Treatment Findings (*n* = 11)

Variables	*Median* (*range*) or *n* (%)
**Treatment Feedback**	
**Client Satisfaction Questionnaire** (mean score: 1–4)	3.75 (2.63–4.00)
**Plan to continue to utilize the skills learned** (1 = *No, not at all*; 10 = *Yes, definitely*)	9 (5–10)
**Helpfulness of mindfulness practice, overall** (1 = *extremely unhelpful*; 6 = *extremely helpful*)	5 (1–6)
**I like the mindfulness practice, overall** (1 = *extremely dislike*; 6 = *extremely like*)	6 (2–6)
**Usability of App and Feedback**	
**System Usability Scale Score** (0–100)	92.50 (22.50–97.50)
**Ease of using the app** (1 = *very difficult*; 6 = *very easy*)	
At the beginning of the study	6 (3–6)
By the end of the study	6 (5–6)
**Ease of instructions for getting started with the app** (1 = *very difficult*; 6 = *very easy*)	6 (4–6)
**Would recommend the app to a friend/family** (1 = *not at all*; 6 = *definitely recommend*)	6 (4–6)
**Technology Acceptance Model (TAM): Perceived usefulness** (1 = *strongly disagree*; 4 = *neutral*; 7 = *strongly agree*)	
Learn mindfulness practice	6 (1–7)
Practice mindfulness daily	6 (1–7)
Cope with cigarette cravings	5 (1–7)
Cope with stress	5 (1–7)
Cope with pain	4 (1–7)
Cope with fatigue	4 (1–7)
Cope with worries about cancer (e.g., recurrence)	4 (1–7)
**TAM: Perceived ease of use**	
Found easy to use the study app to practice mindfulness	6 (1–7)
Found easy to navigate to find the on-demand content	6 (1–7)
**Barriers using the app**^**a**^	
No barriers	10 (90.91%)
Needing to attend to responsibilities (e.g., work, family)	1 (9.09%)
**App Engagement Results**	
**Number of participants who received app engagement reminders after 2 consecutive days of no engagement**	8 (72.73%)
Number of participants who received more than 1 reminder	4 (36.36%)
**Overall app use over 4 weeks (*Mean, SD, range*)**	
Number of weeks	2.31 (1.12, 1–4)
Number of days per week	4.47 (2.21, 1–7)
**EMA completion rate (completed/prompted)**	
Total over 4 weeks	262/1213 (21.60%)
Week 1	77/289 (26.64%)
Week 2	74/308 (24.03%)
Week 3	56/308 (18.18%)
Week 4	55/308 (17.86%)
**Proportion of EMA responses with ≥ 4**	
Total EMA responses ≥ 4/total completed EMA	198/207 (95.65%)
**Proportion of mindful skill topic areas with EMA responses with ≥ 4 (Trigger topic/total EMA responses ≥ 4)**	
Topic 1: cancer	95/198 (47.98%)
Topic 2: craving	13/198 (6.57%)
Topic 3: negative affect (NA)	0/198 (0.00%)
Topic 4: cancer & craving	48/198 (24.24%)
Topic 5: craving & NA	4/198 (2.02%)
Topic 6: cancer & NA	13/198 (6.57%)
Topic 7: cancer & craving & NA	25/198 (12.63%)
**EMI completion rate (completed/prompted)**	
Among displayed EMIs^b^	197/197 (100.00%)
**Follow-Up EMA completion rate (completed/prompted)**	
Total^c^	196/198 (98.99%)
**Random mindful skills completion rate (completed/prompted)**	
Total	112/458 (24.45%)
**Daily meditation completion rate over 4 weeks**	
Practiced ≥ 10 minutes/meditations selected^d^	58/114 (50.88%)
Inquiry (completed/prompted)	91/114 (79.82%)
**On-demand mindful skills completion rate (completed)**	
Total practiced over 4 weeks	47
**On-demand meditation over 4 weeks**	
**Selection rate by length**	
Length selection: 5–6 minutes meditations	32/51 (62.75%)
Length selection: 10–15 minutes meditations	19/51 (37.25%)
**Completion rate**	
Practiced/5–6 minutes meditations selected^d^	25/32 (78.13%)
Practiced/10–15 minutes meditations selected^d^	17/19 (89.46%)
Inquiry (completed/prompted)	42/51 (82.35%)
**Smoking Variables at EOT**	
**Heaviness of smoking index**	1 (0–3)
**Number of days smoked in the past week**	
0 days	2 (18.18%)
1–5 days	5 (45.45%)
7 days	4 (36.36%)
**Number of cigarettes when smoked in the past week (*n* = 9)**	3 (2–15)

^a^*n* = 1 selected “other” and reported trouble accessing the app.

^b^The denominator excludes 1 case which had missingness in three EMA items that trigger EMI (198–1 = 197).

^c^The completed number includes 1 incidence which the three EMA items were missing.

^d^The cutoff was based on the shortest duration of a meditation among the meditations available in the corresponding section.

##### Feedback on the study app: Usability

The perceived usability of the app, as indexed by the SUS, was very high. Participants found the app easy to use at both the beginning and the end of the study, found the instructions for getting started with the app easy to understand, and would recommend the app to someone in need of similar help. Perceived usefulness, as assessed by the modified TAM measures, indicated that using the app helped to learn and practice mindfulness and to cope with cravings and stress, whereas the perceived helpfulness of using the app to cope with pain, fatigue, and worries about cancer was moderate. Over 50.00% of participants reported they would like fewer EMAs and found the EMAs arrived at inconvenient times.

##### Open-ended interview

Most participants, including those who considered themselves not “tech-savvy”, found the smartphone app easy to use and navigate. As one participant put it, the app was “as simple as can be,” and the participant also noted that the download process was uncomplicated. Many suggested reducing the frequency of overall notifications and length of EMAs, recommending the capability to customize the timing of the notifications to better align with their daily activities to practice mindful skills and meditations. Many liked the availability of different meditation lengths and on-demand meditations. Regarding mindful skills, participants were mixed regarding mindful skills related to fear of cancer recurrence, with one participant suggesting removing it because it becomes a reminder of cancer, causing more stress. Many participants liked the “human aspect” of the counseling sessions and found having flexibility between Zoom and phone was helpful when having technical difficulties. Online Resource 5 presents representative quotes.

#### Other Outcomes

##### Counseling Feedback

On the EOT survey, participants found the counseling sessions helpful and using phone/Zoom convenient (Online Resource 4).

##### App Engagement

Table 3 shows the overall engagement statistics. Overall, participants used the app for an average of 14.64 days per person over four weeks (*SD* = 9.49, *range* = 2–28). All participants completed at least 2 EMAs with an average completion rate of 21.56% of the pushed EMAs per person (*M* = 23.81, *SD* = 33.37; *range* = 2–92). The EMA completion rate gradually decreased over time. Among the completed EMAs over the first 3 weeks when EMIs were active, the overwhelming majority met the condition to trigger an EMI (EMA responses with ≥ 4; *n* = 198; 95.65%). The top 3 EMI mindful skill topics triggered were cancer (47.98%), the combination of cancer and craving (24.24%), and the combination of cancer, craving, and negative affect (12.63%). Among the 198 EMA responses with ≥ 4, there was 1 occasion when the EMI was not displayed due to no response to 3 EMA items that trigger EMI. A total of 197 EMIs were correctly displayed (197/198, 99.49%) and 100% of these were completed. Ten participants (10/11, 90.91%) practiced at least 1 EMI, with an average of 19.70 EMIs per person (*SD* = 24.84, *range* = 1–66). A near-perfect completion rate of the follow-up EMAs was observed.

About one-fourth of the prompted random mindful skills (delivered for the first 3 weeks) were completed, with ten participants completing at least one random mindful skill (*M* = 11.20/person, *SD* = 15.14, *range* = 1–40). Regarding the daily meditation practice over 4 weeks, approximately half of the selected meditations were practiced for at least 10 minutes. Nine participants practiced meditation at least once and for at least 10 minutes, with an average completion of 6.44 meditations per person (*SD* = 8.80, *range* = 1–27). The completion rate of the inquiry items was high. Lastly, all participants practiced on-demand mindful skills at least once, and eight participants (8/11, 72.73%) engaged in at least one on-demand meditation. Meditations of 5–6 minutes in length were more frequently selected.

##### Changes in Smoking Behavior

Self-reported seven-day PPA was 18.18% (*n* = 2) at EOT. Among the 9 participants who continued to smoke, the median CPD numerically decreased from 5 (*range* = 2–20) to 3 (*range* = 2–15).

### Discussion

The usability of the app in Study 2 was supported by high ratings on the SUS, as well as perceived usefulness and ease of use. Treatment satisfaction, as indexed by the CSQ, and the retention rate at EOT were also very high. As evidenced in EOT interview findings, the majority of participants found the app easy to use and navigate, including the process of downloading and installing it, even among those who considered themselves less experienced with technology. Usability is regarded as one of the key considerations in developing digital health interventions in various theoretical models (e.g., Efficiency Model of Support, Schueller et al., 2017; Technology Acceptance Model, Davis, 1985, 1989; Lewis & Sauro, 2009). The observed high usability, supported by both quantitative and qualitative evidence, justified the next iteration of intervention development aimed at testing its feasibility and acceptability.

Despite the high usability rating, retention rate, and treatment satisfaction, app engagement was modest. Specifically, the completion rate of EMAs was lower than those reported in the broader mindfulness-based mHealth smoking cessation literature (e.g., ~ 50.00–90.00%; Garrison et al., 2020; Yang et al., 2023), although the overall rate of app use, indicated by the number of weeks and days, was comparable to previous studies (~ 50.00%; Garrison et al., 2020; Minami et al., 2022). There are at least four explanations. First, EMA completion was not compensated due to its potential link to the real-time intervention, EMI. In contrast, mindfulness-based mHealth studies for smoking cessation do typically compensate participants for EMA/daily survey completion (Black & Kirkpatrick, 2023; Garrison et al., 2020; Minami et al., 2022; Yang et al., 2023) and sometimes for completing mindfulness practice (Black & Kirkpatrick, 2023). Our low completion rate may represent real-world engagement without compensation, in particular among cancer survivors (e.g., Paterson et al., 2023). Second, our sample consisting of cancer survivors differed from those in the extant mindfulness-based mHealth cessation literature (e.g., mood disorders, Minami et al., 2022; the general population, Black & Kirkpatrick, 2023; Garrison et al., 2020; Yang et al., 2023), thus, our results may not be directly comparable. Third, participants may have missed the EMAs because each EMA notification expired after 90 minutes and was separated by a minimum interval of 2 h, allowing the next EMA notification to be scheduled for display. However, the high completion rates for the EMIs and follow-up EMAs/inquiry demonstrated that once participants started using the app (e.g., EMA, mindfulness practice), subsequent activities were highly likely to be completed, aligning with previous findings (Horvath et al., 2024; Yang et al., 2023). Lastly, it is possible that the number of daily notifications was perceived as burdensome to some participants. This may have contributed to modest engagement, although the app was perceived as easy to use and learn, and the overall treatment was rated as satisfactory. Participants received a total of 7 notifications per day during the first 3 weeks while the EMI was active. This aligns with key participant feedback suggesting to reduce the frequency of notifications.

## General Discussion

The current paper describes the development and usability of a smartphone app that delivered a mindfulness-based EMI for smoking cessation, addressing both general and cancer-specific risk factors for smoking relapse among cancer survivors. Through an iterative process, we developed a prototype smartphone app by incorporating feedback from cancer survivors and demonstrated its usability, consistent with the goal of the Accelerated Creation-to-Sustainment (ACTS) model’s Create phase (Mohr et al., 2017).

The innovative design of the current study led to several preliminary novel findings. Notably, cancer-related difficulties emerged as a potential key relapse vulnerability among cancer survivors. Negative affect items alone never met the EMI trigger criteria of ≥ 4. Similarly, the craving item alone was one of the least frequent trigger topics. Both constructs became trigger topics only when combined with cancer-related difficulties. The qualitative results from Study 1 showed that cancer survivors did not explicitly connect their smoking to cancer-related difficulties, although they acknowledged their potential contribution. Although the literature has documented cancer-related difficulties as relapse vulnerability factors in longitudinal (pain severity, Cooley et al., 2009; fear of cancer recurrence, Simmons et al., 2013), cross-sectional (e.g., fatigue, Gritz et al., 1999), and qualitative studies (stress from cancer diagnosis, Berg et al., 2013; Wells et al., 2017), the systematic integration of cancer-specific risk factors into existing mindfulness-based smoking cessation treatments has been almost nonexistent (e.g., Charlot et al., 2019; Jackson et al., 2024). Evidence shows that fear of cancer recurrence, pain, and fatigue influence one another in a bidirectional manner among cancer survivors (Gnall et al., 2024; Trudel et al., 2024). Our findings further imply that smoking behaviors may be intertwined with these factors, complicating efforts to quit smoking in this population. These results may partially explain the high relapse rate, highlighting the need to address cancer-related difficulties in smoking cessation interventions. Finally, participants seemed to prefer shorter meditations over longer ones and interview findings indicated that participants appreciated having a choice in the length and timing using the on-demand feature. Previous mindfulness-based mHealth cessation studies provided the within-device formal meditations of relatively fixed durations (5–7 minutes, Minami et al., 2022) or did not report the durations of the meditations and engagement per duration (Black & Kirkpatrick, 2023; Garrison et al., 2020). Our findings may be useful for considering engagement strategies for formal meditation.

Several strengths of the current study include participants with diverse clinical characteristics, high app usability and acceptability, excellent retention rate, and rigorous app development via iterative design with direct user input. In particular, our thorough reporting of app engagement and use data could contribute to the growing need for standard reporting of app engagement to enable comparison of study findings across mHealth studies with heterogeneous designs (Horvath et al., 2024; Torous et al., 2020).

### Limitations and Future Directions

The current study is not without limitations. Our findings should be interpreted with caution, given the small sample size and lack of a comparison arm. Smoking status was only assessed at baseline and at the end of treatment because the primary purpose of the study was usability testing. The intervention may not fully meet the needs of cancer survivors with heterogeneous treatment histories, symptom burden, aftereffects of various surgeries, and psychosocial context. Lastly, app engagement may have been impacted by factors such as cancer-related symptoms (e.g., pain, fatigue, sleep disturbance), mood (e.g., depression), or even surgical history. Given the small sample size, we decided not to examine these factors as related to app engagement. Future iterations of our intervention would benefit from considering these constructs, given their relevance to this population.

Next steps will include updating the app by incorporating participants’ feedback. Given the observed gradual decrease in app engagement over time, we will add one additional brief weekly counseling session between QD and the end of the third week. This strategy follows the Efficiency Model of Support (Schueller et al., 2017), which recommends human support as an engagement strategy in digital health. The language of mindful skills used in the fear of cancer recurrence category will be modified to be less explicit about cancer. The frequency of EMA notifications will be reduced because the majority of completed EMAs met the EMI trigger criteria, and participants recommended reducing the frequency.

To the best of our knowledge, the current study is among the first to develop and demonstrate the usability of an app that provides a mindfulness-based EMI for smoking cessation among cancer survivors. Notably, this intervention is the only study that addressed both general and cancer-specific relapse risks in real time. If found feasible, our intervention will contribute to improving cancer outcomes and the overall quality of life among cancer survivors.

## Supplementary Information

Below is the link to the electronic supplementary material.

Supplementary file1 (PDF 274 kb)

Supplementary file2 (PDF 313 kb)

Supplementary file3 (PDF 389 kb)

Supplementary file4 (PDF 317 kb)

Supplementary file5 (PDF 267 kb)

## Data Availability

Data, materials, and code will be available upon the establishment of Data Use Agreements. Raw verbatim data will not be available for ethical reasons.

## Code Availability

Data, materials, and code will be available upon the establishment of Data Use Agreements. Raw verbatim data will not be available for ethical reasons.

## Declarations

### Use of Artificial Intelligence Statement

Institution–licensed Microsoft 365 Copilot and Grammarly were used to check grammar and correct typographical errors.

## References

[CR1] Alboksmaty, A., Agaku, I. T., Odani, S., & Filippidis, F. T. (2019). Prevalence and determinants of cigarette smoking relapse among US adult smokers: A longitudinal study. *BMJ Open,**9*(11), e031676. 10.1136/bmjopen-2019-031676 31772095 10.1136/bmjopen-2019-031676 PMC6886963

[CR2] Andersen, B. L., Kiecolt-Glaser, J. K., & Glaser, R. (1994). A biobehavioral model of cancer stress and disease course. *American Psychologist,**49*(5), 389–404.10.1037/0003-066X.49.5.389 8024167 10.1037//0003-066x.49.5.389 PMC2719972

[CR3] Berg, C. J., Thomas, A. N., Mertens, A. C., Schauer, G. L., Pinsker, E. A., Ahluwalia, J. S., & Khuri, F. R. (2013). Correlates of continued smoking versus cessation among survivors of smoking‐related cancers. *Psycho-Oncology,**22*(4), 799–806. 10.1002/pon.3077 22488864 10.1002/pon.3077 PMC3425712

[CR4] Biener, L., & Abrams, D. B. (1991). The contemplation ladder: Validation of a measure of readiness to consider smoking cessation. *Health Psychology, 10*(5), 360–365. 10.1037/0278-6133.10.5.360 1935872 10.1037//0278-6133.10.5.360

[CR5] Black, D. S., & Kirkpatrick, M. G. (2023). Effect of a mindfulness training app on a cigarette quit attempt: An investigator-blinded, 58-county randomized controlled trial. *JNCI Cancer Spectrum,**7*(6), pkad095. 10.1093/jncics/pkad095 37951593 10.1093/jncics/pkad095 PMC10715839

[CR6] Borland, R., Yong, H.-H., O’Connor, R., Hyland, A., & Thompson, M. (2010). The reliability and predictive validity of the Heaviness of Smoking Index and its two components: Findings from the International Tobacco Control Four Country study. *Nicotine & Tobacco Research,**12*(suppl 1), S45–S50. 10.1093/ntr/ntq038 20889480 10.1093/ntr/ntq038 PMC3307335

[CR7] Bowen, S., Chawla, N., Grow, J., & Marlatt, A. (2021). *Mindfulness-based relapse prevention for addictive behaviors: A clinician’s guide* (2nd ed). Guilford Publications.

[CR8] Brewer, J. A., Mallik, S., Babuscio, T. A., Nich, C., Johnson, H. E., Deleone, C. M., Minnix-Cotton, C. A., Byrne, S. A., Kober, H., & Weinstein, A. J. (2011). Mindfulness training for smoking cessation: Results from a randomized controlled trial. *Drug and Alcohol Dependence,**119*(1–2), 72–80. 10.1016/j.drugalcdep.2011.05.027 21723049 10.1016/j.drugalcdep.2011.05.027 PMC3191261

[CR9] Businelle, M. S., Ma, P., Kendzor, D. E., Frank, S. G., Vidrine, D. J., & Wetter, D. W. (2016). An ecological momentary intervention for smoking cessation: Evaluation of feasibility and effectiveness. *Journal of Medical Internet Research,**18*(12), e321. 10.2196/jmir.6058 27956375 10.2196/jmir.6058 PMC5187451

[CR10] Carlson, L. E., & Speca, M. (2011). *Mindfulness-based cancer recovery*. New Harbinger Publications.

[CR11] Carlson, L. E., Ismaila, N., Addington, E. L., Asher, G. N., Atreya, C., Balneaves, L. G., Bradt, J., Fuller-Shavel, N., Goodman, J., & Hoffman, C. J. (2023). Integrative oncology care of symptoms of anxiety and depression in adults with cancer: Society for integrative oncology–ASCO guideline. *Journal of Clinical Oncology,**41*(28), 4562–4591. 10.1200/JCO.23.00857 37582238 10.1200/JCO.23.00857

[CR12] Charlot, M., D’Amico, S., Luo, M., Gemei, A., Kathuria, H., & Gardiner, P. (2019). Feasibility and acceptability of mindfulness-based group visits for smoking cessation in low-socioeconomic status and minority smokers with cancer. *Journal of Alternative and Complementary Medicine,**25*(7), 762–769. 10.1089/acm.2019.0016 31314565 10.1089/acm.2019.0016 PMC9889013

[CR13] Cleeland, C. (1989). Measurement of pain by subjective report. *Advances in Pain Research and Therapy,**12*, 391–403.

[CR14] Cooley, M. E., Sarna, L., Kotlerman, J., Lukanich, J. M., Jaklitsch, M., Green, S. B., & Bueno, R. (2009). Smoking cessation is challenging even for patients recovering from lung cancer surgery with curative intent. *Lung Cancer,**66*(2), 218–225. 10.1016/j.lungcan.2009.01.021 19321223 10.1016/j.lungcan.2009.01.021 PMC3805262

[CR15] Crane, R., Brewer, J., Feldman, C., Kabat-Zinn, J., Santorelli, S., Williams, J., & Kuyken, W. (2017). What defines mindfulness-based programs? The warp and the weft. *Psychological Medicine,**47*(6), 990–999. 10.1017/S0033291716003317 28031068 10.1017/S0033291716003317

[CR16] Custers, J. A., Kwakkenbos, L., van de Wal, M., Prins, J. B., & Thewes, B. (2018). Re‐validation and screening capacity of the 6‐item version of the cancer worry scale. *Psycho-Oncology,**27*(11), 2609–2615. 10.1002/pon.4782 29843189 10.1002/pon.4782

[CR17] Davis, F. D. (1989). Perceived usefulness, perceived ease of use, and user acceptance of information technology. *MIS Quarterly,**13*, 319–340. 10.2307/249008

[CR18] Davis, J. M., Goldberg, S. B., Anderson, M. C., Manley, A. R., Smith, S. S., & Baker, T. B. (2014). Randomized trial on mindfulness training for smokers targeted to a disadvantaged population. *Substance Use & Misuse,**49*(5), 571–585. 10.3109/10826084.2013.770025 24611852 10.3109/10826084.2013.770025 PMC3955013

[CR19] Davis, F. D. (1985). *A technology acceptance model for empirically testing new end-user information systems: Theory and results* [Doctoral dissertation, Massachusetts Institute of Technology].

[CR20] de Weiss Souza, I. C., Kozasa, E. H., Bowen, S., Richter, K. P., Sartes, L. M. A., Colugnati, F. A. B., & Noto, A. R. (2020). Effectiveness of mindfulness-based relapse prevention program as an adjunct to the standard treatment for smoking: A pragmatic design pilot study. *Nicotine & Tobacco Research,**22*(9), 1605–1613. 10.1093/ntr/ntaa057 32222767 10.1093/ntr/ntaa057

[CR21] Faulkner, L. (2003). Beyond the five-user assumption: Benefits of increased sample sizes in usability testing. *Behavior Research Methods, Instruments, & Computers,**35*(3), 379–383. 10.3758/bf03195514 10.3758/bf03195514 14587545

[CR22] Feuer, Z., Michael, J., Morton, E., Matulewicz, R. S., Sheeran, P., Shoenbill, K., Goldstein, A., Sherman, S., & Bjurlin, M. A. (2022). Systematic review of smoking relapse rates among cancer survivors who quit at the time of cancer diagnosis. *Cancer Epidemiology,**80*, 102237. 10.1016/j.canep.2022.102237 35988307 10.1016/j.canep.2022.102237 PMC10363369

[CR23] Florou, A. N., Gkiozos, I. C., Tsagouli, S. K., Souliotis, K. N., & Syrigos, K. N. (2014). Clinical significance of smoking cessation in subjects with cancer: A 30-year review. *Respiratory Care,**59*(12), 1924–1936. 10.4187/respcare.02559 25185148 10.4187/respcare.02559

[CR24] Gale, R. C., Wu, J., Erhardt, T., Bounthavong, M., Reardon, C. M., Damschroder, L. J., & Midboe, A. M. (2019). Comparison of rapid vs in-depth qualitative analytic methods from a process evaluation of academic detailing in the Veterans Health Administration. *Implementation Science,**14*, 1–12. 10.1186/s13012-019-0853-y 30709368 10.1186/s13012-019-0853-y PMC6359833

[CR25] Garrison, K. A., Pal, P., O’Malley, S. S., Pittman, B. P., Gueorguieva, R., Rojiani, R., Scheinost, D., Dallery, J., & Brewer, J. A. (2020). Craving to quit: A randomized controlled trial of smartphone app–based mindfulness training for smoking cessation. *Nicotine & Tobacco Research,**22*(3), 324–331. 10.1093/ntr/nty126 29917096 10.1093/ntr/nty126 PMC7297096

[CR26] Gnall, K. E., Emrich, M., Magin, Z. E., Park, C. L., Bellizzi, K. M., & Sanft, T. (2024). Anxiety and fear of cancer recurrence as predictors of subsequent pain interference in early cancer survivorship: Exploring the moderating roles of cognitive and emotional factors. *Journal of Behavioral Medicine,**47*(6), 980–993. 10.1007/s10865-024-00506-1 39110352 10.1007/s10865-024-00506-1

[CR27] Gritz, E. R., Schacherer, C., Koehly, L., Nielsen, I. R., & Abemayor, E. (1999). Smoking withdrawal and relapse in head and neck cancer patients. *Head & Neck,**21*(5), 420–427. 10.1002/(sici)1097-0347(199908)21:53.0.co;2-u 10402522 10.1002/(sici)1097-0347(199908)21:5<420::aid-hed7>3.0.co;2-u

[CR28] Gritz, E. R., Talluri, R., Domgue, J. F., Tami-Maury, I., & Shete, S. (2020). Smoking behaviors in survivors of smoking-related and non–smoking-related cancers. *JAMA Network Open,**3*(7), e209072. 10.1001/jamanetworkopen.2020.9072 32614423 10.1001/jamanetworkopen.2020.9072 PMC7333020

[CR29] Guest, G., MacQueen, K. M., & Namey, E. E. (2011). *Applied thematic analysis*. Sage Publications.

[CR30] Guimond, A.-J., Croteau, V. A., Savard, M.-H., Bernard, P., Ivers, H., & Savard, J. (2017). Predictors of smoking cessation and relapse in cancer patients and effect on psychological variables: An 18-month observational study. *Annals of Behavioral Medicine,**51*(1), 117–127. 10.1007/s12160-016-9834-4 27670773 10.1007/s12160-016-9834-4

[CR31] Heatherton, T. F., Kozlowski, L. T., Frecker, R. C., Rickert, W., & Robinson, J. (1989). Measuring the heaviness of smoking: Using self‐reported time to the first cigarette of the day and number of cigarettes smoked per day. *British Journal of Addiction,**84*(7), 791–800. 10.1111/j.1360-0443.1989.tb03059.x 2758152 10.1111/j.1360-0443.1989.tb03059.x

[CR32] Hébert, E. T., Ra, C. K., Alexander, A. C., Helt, A., Moisiuc, R., Kendzor, D. E., Vidrine, D. J., Funk-Lawler, R. K., & Businelle, M. S. (2020). A mobile just-in-time adaptive intervention for smoking cessation: Pilot randomized controlled trial. *Journal of Medical Internet Research,**22*(3), e16907. 10.2196/16907 32149716 10.2196/16907 PMC7091024

[CR33] Horvath, M., Pittman, B., O’Malley, S. S., Grutman, A., Khan, N., Gueorguieva, R., Brewer, J. A., & Garrison, K. A. (2024). Smartband-based smoking detection and real-time brief mindfulness intervention: Findings from a feasibility clinical trial. *Annals of Medicine,**56*(1), 2352803. 10.1080/07853890.2024.2352803 38823419 10.1080/07853890.2024.2352803 PMC11146247

[CR34] Jackson, R. W., Cao-Nasalga, A., Chieng, A., Pirkl, A., Jagielo, A. D., Xu, C., Goldenhersch, E., Rosencovich, N., Waitman, C., & Prochaska, J. J. (2024). Adding virtual reality mindful exposure therapy to a cancer center’s tobacco treatment offerings: Feasibility and acceptability single-group pilot study. *JMIR Formative Research,**8*(1), e54817. 10.2196/54817 39042439 10.2196/54817 PMC11303906

[CR35] Jassem, J. (2019). Tobacco smoking after diagnosis of cancer: Clinical aspects. *Translational Lung Cancer Research,**8*(Suppl 1), S50–S58. 10.21037/tlcr.2019.04.01 31211105 10.21037/tlcr.2019.04.01 PMC6546630

[CR36] Kabat-Zinn, J. (2013). *Full catastrophe living* (Revised ed). Bantam.

[CR37] Kabat-Zinn, J. (2023). *Mindfulness meditation for pain relief: Practices to reclaim your body and your life*. Sounds True.

[CR38] Kober, H., Brewer, J. A., Height, K. L., & Sinha, R. (2017). Neural stress reactivity relates to smoking outcomes and differentiates between mindfulness and cognitive-behavioral treatments. *NeuroImage,**151*, 4–13. 10.1016/j.neuroimage.2016.09.042 27693614 10.1016/j.neuroimage.2016.09.042 PMC5373945

[CR39] Kroenke, K., Strine, T. W., Spitzer, R. L., Williams, J. B., Berry, J. T., & Mokdad, A. H. (2009). The PHQ-8 as a measure of current depression in the general population. *Journal of Affective Disorders,**114*(1–3), 163–173. 10.1016/j.jad.2008.06.026 18752852 10.1016/j.jad.2008.06.026

[CR40] Land, S. R., Warren, G. W., Crafts, J. L., Hatsukami, D. K., Ostroff, J. S., Willis, G. B., Chollette, V. Y., Mitchell, S. A., Folz, J. N., & Gulley, J. L. (2016). Cognitive testing of tobacco use items for administration to patients with cancer and cancer survivors in clinical research. *Cancer,**122*(11), 1728–1734. 10.1002/cncr.29964 27019325 10.1002/cncr.29964 PMC5523930

[CR41] Larsen, D. L., Attkisson, C. C., Hargreaves, W. A., & Nguyen, T. D. (1979). Assessment of client/patient satisfaction: Development of a general scale. *Evaluation and Program Planning,**2*(3), 197–207. 10.1016/0149-7189(79)90094-6 10245370 10.1016/0149-7189(79)90094-6

[CR42] Lehrhaupt, L., & Meibert, P. (2017). *Mindfulness-based stress reduction: The MBSR program for enhancing health and vitality*. New World Library.

[CR43] Lewis, J., & Sauro, J. (2009). *The factor structure of the System Usability Scale.* Proceedings of the 1st International Conference on Human Centered Design: Held as Part of HCI International 2009, San Diego, CA.

[CR44] Linehan, M. M. (2014). *DBT® skills training manual* (2nd ed). Guilford Press.

[CR45] Lumivero. (2025). *NVivo* (Version 12) [Computer software]. https://lumivero.com/products/nvivo/

[CR46] McHugh, M. L. (2012). Interrater reliability: The kappa statistic. *Biochemia Medica,**22*(3), 276–282. 10.11613/BM.2012.031 23092060 PMC3900052

[CR47] Mendoza, T. R., Wang, X. S., Cleeland, C. S., Morrissey, M., Johnson, B. A., Wendt, J. K., & Huber, S. L. (1999). The rapid assessment of fatigue severity in cancer patients: Use of the Brief Fatigue Inventory. *Cancer,**85*(5), 1186–1196. 10091805 10.1002/(sici)1097-0142(19990301)85:5<1186::aid-cncr24>3.0.co;2-n

[CR48] Metricwire Inc. *Catalyst By MetricWire*. https://metricwire.com/

[CR49] Minami, H., Nahvi, S., Arnsten, J. H., Brinkman, H. R., Rivera-Mindt, M., Wetter, D. W., Bloom, E. L., Price, L. H., Richman, E. K., & Betzler, T. F. (2022). A pilot randomized controlled trial of smartphone-assisted mindfulness-based intervention with contingency management for smokers with mood disorders. *Experimental and Clinical Psychopharmacology,**30*(5), 653–665. 10.1037/pha0000506 34291992 10.1037/pha0000506

[CR50] Mohr, D. C., Lyon, A. R., Lattie, E. G., Reddy, M., & Schueller, S. M. (2017). Accelerating digital mental health research from early design and creation to successful implementation and sustainment. *Journal of Medical Internet Research,**19*(5), e153. 10.2196/jmir.7725 28490417 10.2196/jmir.7725 PMC5443926

[CR51] National Cancer Institute. (2008). *Clearing the air: Quit smoking today*. NIH Publication.

[CR52] NCI-AACR Cancer Patient Tobacco Use Assessment Task Force. (2016). *C-TUQ user manual: Cancer Patient Tobacco Use Questionnaire*.

[CR53] Paterson, C., Armitage, L., & Turner, M. (2023). Current landscape of ecological momentary assessment (real-time data) methodology in cancer research: A systematic review. *Seminars in Oncology Nursing,**39*(6), 151514. 10.1016/j.soncn.2023.151514 37865555 10.1016/j.soncn.2023.151514

[CR54] Roos, C., Stein, E., Kirouac, M., Bowen, S., & Witkiewitz, K. (n.d.). *A clinician’s guide to mindfulness-Based Relapse Prevention Rolling Admission (MBRP-RA)*. https://www.mindfulrp.com/for-clinicians 10.1007/s12671-018-1054-5 PMC6660179 31354877

[CR55] Ruscio, A. C., Muench, C., Brede, E., & Waters, A. J. (2016). Effect of brief mindfulness practice on self-reported affect, craving, and smoking: A pilot randomized controlled trial using ecological momentary assessment. *Nicotine & Tobacco Research,**18*(1), 64–73. 10.1093/ntr/ntv074 25863520 10.1093/ntr/ntv074

[CR56] Schueller, S. M., Tomasino, K. N., & Mohr, D. C. (2017). Integrating human support into behavioral intervention technologies: The efficiency model of support. *Clinical Psychology: Science and Practice,**24*(1), 27–45. 10.1111/cpsp.12173

[CR57] Sheeran, P., Jones, K., Avishai, A., Symes, Y. R., Abraham, C., Miles, E., Wright, C. E., Mayer, D. K., & Ribisl, K. M. (2019). What works in smoking cessation interventions for cancer survivors? A meta-analysis. *Health Psychology,**38*(10), 855–865. 10.1037/hea0000757 31259596 10.1037/hea0000757

[CR58] Shields, P. G., Bierut, L., Arenberg, D., Balis, D., Cinciripini, P. M., Davis, J., Edmondson, D., Feliciano, J., Hitsman, B., & Hudmon, K. S. (2023). Smoking cessation, version 3.2022, NCCN clinical practice guidelines in oncology. *Journal of the National Comprehensive Cancer Network,**21*(3), 297–322. 10.6004/jnccn.2023.0013 36898367 10.6004/jnccn.2023.0013

[CR59] Simmons, V. N., Litvin, E. B., Jacobsen, P. B., Patel, R. D., McCaffrey, J. C., Oliver, J. A., Sutton, S. K., & Brandon, T. H. (2013). Predictors of smoking relapse in patients with thoracic cancer or head and neck cancer. *Cancer,**119*(7), 1420–1427. 10.1002/cncr.27880 23280005 10.1002/cncr.27880 PMC3604135

[CR60] Strauss, A., & Corbin, J. (1998). *Basics of qualitative research techniques*. Sage Publications.

[CR61] Torous, J., Michalak, E. E., & O’Brien, H. L. (2020). Digital health and engagement—Looking behind the measures and methods. *JAMA Network Open,**3*(7), e2010918. 10.1001/jamanetworkopen.2020.10918 32678446 10.1001/jamanetworkopen.2020.10918

[CR62] Trudel, G., Lebel, S., Stephens, R. L., Leclair, C. S., Leach, C. R., & Westmaas, J. L. (2024). Afraid and tired: A longitudinal study of the relationship between cancer‐related fatigue and fear of cancer recurrence in long‐term cancer survivors. *Cancer Medicine,**13*(11), Article e7313. 10.1002/cam4.7313 38845458 10.1002/cam4.7313 PMC11157147

[CR63] U.S. National Cancer Institute. (2022). *Treating smoking in cancer patients: An essential component of cancer care*. U.S. Department of Health and Human Services, National Institutes of Health, National Cancer Institute.

[CR64] U.S. National Cancer Institute. Retrieved February 28 from https://cancercontrol.cancer.gov/ocs/definitions#statistics

[CR65] US Department of Health and Human Services. (2020). *Smoking cessation: A report of the Surgeon General - Executive summary*. U.S. Department of Health and Human Services, Centers for Disease Control and Prevention, National Center for Chronic Disease Prevention and Health Promotion, Office on Smoking and Health.

[CR66] Vidrine, J. I., Spears, C. A., Heppner, W. L., Reitzel, L. R., Marcus, M. T., Cinciripini, P. M., Waters, A. J., Li, Y., Nguyen, N. T. T., & Cao, Y. (2016). Efficacy of mindfulness-based addiction treatment (MBAT) for smoking cessation and lapse recovery: A randomized clinical trial. *Journal of Consulting and Clinical Psychology,**84*(9), 824–838. 10.1037/ccp0000117 27213492 10.1037/ccp0000117 PMC5061584

[CR67] Vinci, C., Malkhasyan, L., Simmons, V. N., & Correa-Fernandez, V. (2020). The relationship of mindfulness and mindfulness-related practices with alcohol use among Hispanics/Latinx. *Psychiatry Research,**285*, 112774. 10.1016/j.psychres.2020.112774 32035378 10.1016/j.psychres.2020.112774

[CR68] Vinci, C., Sutton, S. K., Yang, M., Jones, S. R., Kumar, S., & Wetter, D. W. (2025). Proximal effects of a just-in-time adaptive intervention for smoking cessation with wearable sensors: A micro-randomized trial. *JMIR Mhealth Uhealth,**13*, e55379. 10.2196/55379 40106803 10.2196/55379 PMC11966069

[CR69] Warren, G. W., Cartmell, K. B., Garrett-Mayer, E., Salloum, R. G., & Cummings, K. M. (2019). Attributable failure of first-line cancer treatment and incremental costs associated with smoking by patients with cancer. *JAMA Network Open,**2*(4), e191703. 10.1001/jamanetworkopen.2019.1703 30951159 10.1001/jamanetworkopen.2019.1703 PMC6450325

[CR70] Wells, M., Aitchison, P., Harris, F., Ozakinci, G., Radley, A., Bauld, L., Entwistle, V., Munro, A., Haw, S., & Culbard, B. (2017). Barriers and facilitators to smoking cessation in a cancer context: A qualitative study of patient, family and professional views. *BMC Cancer,**17*, 348. 10.1186/s12885-017-3344-z 28526000 10.1186/s12885-017-3344-z PMC5438552

[CR71] Yang, M. J., Yepez, V. V., Brandon, K. O., Reblin, M., Pidala, J., Jim, H. S. L., Meyer, J. S., Gore, L. R., Khera, N., Lau, P., Sauls, R. M., Jones, S. R., & Vinci, C. (2022). A mindfulness-based stress management program for caregivers of allogeneic hematopoietic stem cell transplant (HCT) patients: Protocol for a randomized controlled trial. *PLoS ONE,**17*(4), e0266316. 10.1371/journal.pone.0266316 35363799 10.1371/journal.pone.0266316 PMC8975158

[CR72] Yang, M. J., Sutton, S. K., Hernandez, L. M., Jones, S. R., Wetter, D. W., Kumar, S., & Vinci, C. (2023). A mindfulness-based just-in-time adaptive intervention for smoking cessation: Feasibility and acceptability findings. *Addictive Behaviors,**136*, 107467. 10.1016/j.addbeh.2022.107467 36037610 10.1016/j.addbeh.2022.107467 PMC10246550

