# Supplementary material for: Mindfulness-Based Ecological Momentary Intervention for Smoking Cessation to Address Cancer-Related Relapse Risk Factors: Intervention Development and Usability Findings
Source: Mindfulness (N Y). 2026 Mar 9;17(4):1101–19. doi: 10.1007/s12671-026-02775-0 (PMC12971066; doi:10.1007/s12671-026-02775-0)
Supplement: Supplementary file 1 — Supplementary file1 (PDF 274 kb) [file 12671_2026_2775_MOESM1_ESM.pdf]

Online Resource 1. Study 1: Representative Quotes

Theme	Sub-themes and quotes
**1. Perceived usefulness of mindfulness for quitting smoking for cancer survivors**	**Reactions to mindfulness practice**<br><br>*"I was very aware of my head and how bad it hurt. I live in constant pain ever since my very first surgery. But when you said just leave it there, it was kind of recognizing or acknowledging what's happening but then moving on. And I think that was kind of may be the point. … just take pause… feel the feeling but leave it there and move on."* (age 51, female, breast cancer)
	**Perceived usefulness**<br><br>*"… being a cancer survivor, that every time I feel… a pain here or there, my focus goes right to cancer. Do I have cancer again? But if I could meditate and feel maybe what's going on, I could take the focus off of the cancer and back on to just… it's normal old body."* (age 62, female, breast cancer)<br><br>*"… it might be helpful, when feeling stress, instead of picking up a cigarette… I think smoking for me is more of a habit. When I (had) first diagnosis, I smoked more because of stress, I know that."* (age 56, female, breast cancer)
**2. Acceptance of mHealth**	**Perceived usefulness of ecological momentary intervention and app use**<br><br>*"I haven't experienced a lot of pain, but the fatigue I have. I think prompt would help with that because if you're that fatigued and get out, you sometimes need a little push to get back with the program. Get back to trying to get yourself out of that slump."* (age 72, female, lung cancer)
	**Perceived ease of use**<br><br>*"It was pretty straightforward."* (age 75, male, lung cancer)
	**Attitudes toward using an app**<br><br>*"It seems it's a new, something different, something new that doesn't involve physical medication."* (age 62, male, skin cancer)
	**Behavioral intention to use**<br><br>*"I think there should be more help out there to quit this horrible drug [smoking]. … after quitting for a month, I did finally get to the point where they [cigarettes] really stink. … I think this could be very helpful, I really do. I definitely wanna go forward without a doubt, I want to try this."* (age 56, male, lung cancer)
**3. Quitting challenges**	**Prior quitting attempts, relapse, and interests in quitting**<br><br>*"… to just cut it [smoking] like that, I really felt that it was very easy to do, but then… I don't know what happened… really bad, stressful day, and then I smoked. And then one thing leads to another, and then it's just that freaking habit all over again… now it doesn't seem so easy. It's hard [to quit smoking]."* (age 56, male, lung cancer)<br><br>*"I've had three surgeries, and I've had two colostomy bags, and the first one, after my surgery it was such a hard recovery, and I had to have another surgery that following year, so I quit smoking and I recovered much faster than when I'm smoking."* (age 56, female, colon cancer)

	**General smoking vulnerabilities**<br><br>*"I am one of those people that have quit smoking several times. But then, stress just kind of leads me back down that path. I feel like without having, you know, some other technique to try to combat that urge, I'm just gonna continue to keep going down that path."* (age 57, female, gynecological cancer)
	**Cancer-related smoking vulnerabilities**<br><br>*"I actually found myself hiding more since people knew I had cancer, because I felt like they would be judging me. Well, maybe if you didn't smoke you wouldn't have cancer… you know, people do judge you. … I felt like people were like, well, you know because you smoke is why this happened to you, you know, but yet you're going to continue to smoke… I'm gonna cry, but then you get mad at yourself because you can't quit, you know, and it's just a cycle… I want to be over it."* (age 57, female, gynecological cancer)<br><br>*"The shame and the guilt is very high… make it difficult to quit smoking because it [is] a revolving door. … like the hamster in the wheel… just keeps going round and round. … You feel bad that you're smoking and then you smoke. And then you feel bad because you're smoking and then you smoke, it's just like a vicious circle. I had the upper left lung removed and then the treatments of the chemo and stuff. … now we've started the immunotherapy and at this point… there are no cancers, no new cancers. I hope that it stays that way, but the thought [cancer recurrence] is always there you know? Especially with continuing smoking, you know. I feel like an idiot, you know, to be honest, but I'm addicted and it's difficult… it hurts to see it and to know it… I feel powerless."* (age 56, male, lung cancer)
**4. Suggestions for app and intervention content**	**Mindfulness content**<br><br>*"… it was mentioning like people's fear of recurrence and that kind of thing, I would think that's probably pretty high up there on the list of things, because not everybody has pain, not everybody has fatigue, you know. If you were successful in treatment… I think the main thing that's gonna left to me is going to be the worry about the recurrence, so maybe a little focus on that… I mean, everybody would probably feel that I think just about everybody."* (age 62, male, skin cancer)
	**App content**<br><br>*"I would keep the survey as short as possible, if it's gonna be four times a day, somebody would be, "oh tired of doing this," you know, or something, if it becomes too much, too involved."* (age 62, male, skin cancer)
	**Feel and look of app**<br><br>*"I think the reminders is a good thing… just a reminder, like 'hey, we're here! Don't forget to take your five deep breaths,' 'don't forget to take one minute to close your eyes and feel what you're feeling, or feel your body' or whatever."* (age 51, female, breast cancer)
	**Counseling session**<br><br>*"Take into consideration… the specialty of what affects that person, like my illness, they have to take that into consideration, you know… it can't be just a flat thing for the same thing for everybody. It has to be personalized."* (age 74, female, breast cancer)

	**<u>Tips for future patients</u>**<br><br>*"… maybe a thing [notification] pops up and they're in the middle of a meeting at work. Well, I can't fill it out then, so does the little trigger stay on for later and I can do it, or do I just skip that one because another one is coming out? … [tell people] it's here to help you, you need to use it, you should use it. But if you don't or you miss a day, may be you're on vacation for a week… don't be alarmed, it's okay… I would want to put people at ease."* (age 51, female, breast cancer)<br><br>*"Well, I think this interview that we have done was very very helpful. Just let them know that I'm not here to tell you to quit, I'm here to just try and help you become aware of each time you're ready to light up that there you have other options… it was very helpful for me, like you're not here to push it on me that I have to quit smoking tomorrow or you're not telling me that, you know, if I keep doing it, it's gonna kill me, you know. No, people don't want to hear that. They wanna hear that you're going to encourage them to try this instead of that."* (age 62, female, breast cancer)
