# Supplementary material for: Mindfulness-Based Ecological Momentary Intervention for Smoking Cessation to Address Cancer-Related Relapse Risk Factors: Intervention Development and Usability Findings
Source: Mindfulness (N Y). 2026 Mar 9;17(4):1101–19. doi: 10.1007/s12671-026-02775-0 (PMC12971066; doi:10.1007/s12671-026-02775-0)
Supplement: Supplementary file 2 — Supplementary file2 (PDF 313 kb) [file 12671_2026_2775_MOESM2_ESM.pdf]

Online Resource 2. Study 2: Intervention Content and EMA Items

Online Resource 2.1. Meditation

Meditation	Length availability (10-15 minutes or both 5-6/10-15 minutes)	Practiced in counseling session	Timing of availability
Urge surfing	10-15 minutes	Session 1	Week 1
Body scan	Both		
Mindful check-in	Both		
Mindful movement	Both		
Breath meditation	Both	Session 2	Week 2-3
Mindfulness of emotions	Both		
Mindfulness of pain/achiness	Both		
Sitting mediation: sound, breath, sensations, thought	Both		
SOBER breathing space	Both		
Loving kindness meditation	Both	Session 3	Week 4
Values meditation	10-15 minutes		

Online Resource 2.2. Mindful Skills

Mindful skill topic area	Number of skills	EMI category
1. Fear of cancer recurrence	13	Cancer-related
2. Pain	16	Cancer-related
3. Fatigue	10	Cancer-related
4. Self-compassion	11	General
5. Emotions	14	Negative affect
6. Compassionate mindful movement	8	General
7. Awareness of breath	10	General
8. Awareness of thoughts	15	General
9. Awareness of sensations	11	General
10. Acceptance/nonjudging of experience	14	General
11. Loving kindness	10	General
12. Gratitude	14	General

13. Being present with uncertainty	16	General
14. Cravings	31	Cravings
**Total Number of Mindful Skills**	**193**	

Notes. EMI = Ecological Momentary Intervention

Online Resource 2.3. Ecological Momentary Assessment (EMA) Items

EMA Construct	Item	Scale
Mindfulness	Just now, I was perceiving my feelings and thoughts without having to react them.	15-Item Five Facet Mindfulness Questionnaire (Gu et al., 2016)
	Just now, I was "running on automatic" without much awareness of what I was doing.	Mindful Attention Awareness Scale (Brown & Ryan, 2003)
	Just now, I was caught up in my thoughts and feelings.	Decentering (Naragon-Gainey et al., 2023)
Affect	Right now, I feel ashamed.	Positive and Negative Affect Schedule (Thompson, 2007; Watson & Clark, 1994; Watson et al., 1988)
	Right now, I feel calm.	
	Right now, I feel nervous.	
	Right now, I feel happy.	
	Right now, I feel sad.	
	Right now, I feel distressed.	
Craving	Right now, I have an urge to smoke.	Craving (Yang et al., 2022)
Cancer-related	Right now, how concerned are you about the possibility of getting cancer again one day?	Cancer Worry Scale (Harnas et al., 2023)
	Right now, how much fatigue (weariness, tiredness) are you experiencing?	Brief Fatigue Inventory (Mendoza et al., 1999)
	Right now, how much pain do you have?	Brief Pain Inventory (Cleeland, 2009)

Online Resource 2 References

Brown, K. W., & Ryan, R. M. (2003). The benefits of being present: Mindfulness and its role in psychological well-being. *Journal of Personality and Social Psychology*, *84*(4), 822-848. https://doi.org/10.1037/0022-3514.84.4.822

Cleeland, C. S. (2009). The Brief Pain Inventory: User guide. https://www.mdanderson.org/documents/Departments-and-Divisions/Symptom-Research/BPI_UserGuide.pdf

Gu, J., Strauss, C., Crane, C., Barnhofer, T., Karl, A., Cavanagh, K., & Kuyken, W. (2016). Examining the factor structure of the 39-item and 15-item versions of the Five Facet Mindfulness Questionnaire before and after mindfulness-based cognitive therapy for people with recurrent depression. *Psychological Assessment*, *28*(7), 791-802. https://doi.org/10.1037/pas0000263

Harnas, S. J., Booij, S. H., Csorba, I., Nieuwkerk, P. T., Knoop, H., & Braamse, A. M. (2023). Which symptom to address in psychological treatment for cancer survivors when fear of cancer recurrence, depressive symptoms, and cancer-related fatigue co-occur? Exploring the level of agreement between three systematic approaches to select the focus of treatment. *Journal of Cancer Survivorship, 18*(6), 1822-1834. https://doi.org/10.1007/s11764-023-01423-z

Mendoza, T. R., Wang, X. S., Cleeland, C. S., Morrissey, M., Johnson, B. A., Wendt, J. K., & Huber, S. L. (1999). The rapid assessment of fatigue severity in cancer patients: Use of the Brief Fatigue Inventory. *Cancer*, *85*(5), 1186-1196. https://doi.org/10.1002/(sici)1097-0142(19990301)85:5<1186::aid-cncr24>3.0.co;2-n

Naragon-Gainey, K., DeMarree, K. G., Kyron, M. J., McMahon, T. P., Park, J., & Biehler, K. M. (2023). Decentering from emotions in daily life: Dynamic associations with affect, symptoms, and wellbeing. *Clinical Psychological Science*, *11*(5), 841-862. https://doi.org/10.1177/21677026221147262

Thompson, E. R. (2007). Development and validation of an internationally reliable short-form of the positive and negative affect schedule (PANAS). *Journal of Cross-Cultural Psychology*, *38*(2), 227-242. https://doi.org/10.1177/0022022106297301

Watson, D., & Clark, L. A. (1994). The PANAS-X: Manual for the positive and negative affect schedule-expanded form. https://www2.psychology.uiowa.edu/faculty/clark/panas-x.pdf

Watson, D., Clark, L. A., & Tellegen, A. (1988). Development and validation of brief measures of positive and negative affect: The PANAS scales. *Journal of Personality and Social Psychology*, *54*(6), 1063-1070. https://doi.org/10.1037/0022-3514.54.6.1063

Yang, M. J., Brandon, K. O., Sutton, S. K., Kleinjan, M., Hernandez, L. M., Sawyer, L. E., Brandon, T. H., & Vinci, C. (2022). Augmented reality for extinction of cue-provoked urges to smoke: Proof of concept. *Psychology of Addictive Behaviors*, *36*(8), 990-908. https://doi.org/10.1037/adb0000868
