# Supplementary figures and images for: Mindfulness-Based Ecological Momentary Intervention for Smoking Cessation to Address Cancer-Related Relapse Risk Factors: Intervention Development and Usability Findings
Source: Mindfulness (N Y). 2026 Mar 9;17(4):1101–19. doi: 10.1007/s12671-026-02775-0 (PMC12971066; doi:10.1007/s12671-026-02775-0)

### Online Resource 3. Sample Screenshots of the Prototype App and EMI Logic

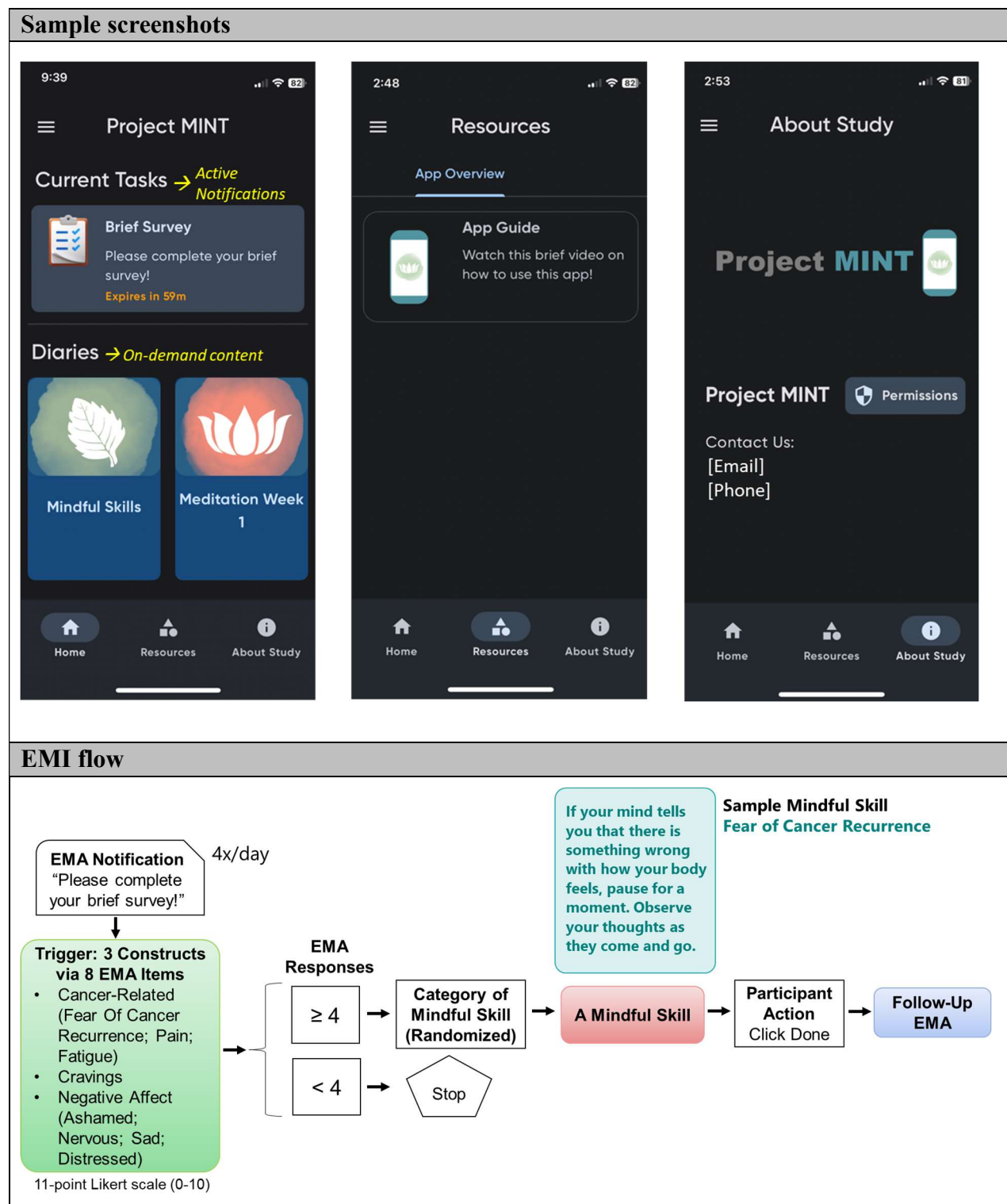

Supplement: Supplementary file 3 — Supplementary file3 (PDF 389 kb) [file 12671_2026_2775_MOESM3_ESM.pdf]
