# Supplementary material for: Mindfulness-Based Ecological Momentary Intervention for Smoking Cessation to Address Cancer-Related Relapse Risk Factors: Intervention Development and Usability Findings
Source: Mindfulness (N Y). 2026 Mar 9;17(4):1101–19. doi: 10.1007/s12671-026-02775-0 (PMC12971066; doi:10.1007/s12671-026-02775-0)
Supplement: Supplementary file 4 — Supplementary file4 (PDF 317 kb) [file 12671_2026_2775_MOESM4_ESM.pdf]

Online Resource 4. Study 2: Additional Survey Results

Variables	*Median* (*range*) or *n* (%)
**Treatment Feedback**	
**Mindfulness practiced helped** (1 = *strongly disagree*; 6 = *strongly agree*)	
Decrease my smoking	6 (3-6)
Cope with my craving	5 (3-6)
Cope with stress	4 (1-6)
Cope with pain[a]	4 (3-6)
Cope with fatigue[b]	4 (2-6)
Cope with worries about cancer[b]	5 (1-6)
**Number of brief mindful skills**	
The number of skills received each day was just right	8 (72.73%)
Would have liked fewer skills each day	3 (27.27%)
**Amount of brief mindful skills sent each day over time**	
Liked the consistent amount of skills	6 (54.55%)
Would have liked more skills	2 (18.18%)
Would have liked fewer skills	3 (27.27%)
**Timing of the brief mindful skills throughout the day**	
Liked the random skills	6 (54.55%)
Sometimes felt arrived at inconvenient times	4 (36.36%)
Often felt arrived at inconvenient times	1 (9.09%)
**Content of the brief mindful skills**	
The variety in content was just right	11 (100.00%)
**Content of the meditation audio recordings**	
Too much variety	1 (9.09%)
The variety in content was just right	9 (81.82%)
Would have liked more variety in content	1 (9.09%)
**Length of meditation audio recordings**	
Each mediation was too long	2 (18.18%)
The length was just right	9 (81.82%)
**Feedback on EMA**	
**Length of brief surveys (i.e., EMA)[c]**	
Would have liked shorter surveys	4 (36.36%)
The length was just right	6 (54.55%)
**Number of brief surveys (i.e., EMA)**	
Would have liked fewer surveys each day	7 (63.64%)
The number of surveys was just right	4 (36.36%)
**Timing of receiving brief surveys (i.e., EMA)**	
The timing was just right	4 (36.36%)
Sometimes felt arrived at inconvenient times	6 (54.55%)
Often felt arrived at inconvenient times	1 (9.09%)
**Feedback on Counseling Sessions**	
**Convenience using phone or Zoom**	10 (5-10)

(1 = *not at all convenient*; 10 = *very convenient*)	
**Length of counseling sessions**	
Too long	3 (27.27%)
Just right	8 (72.73%)
**Number of counseling sessions**	
Would have liked fewer sessions	1 (9.09%)
The number was just right	9 (81.82%)
Would have liked a greater number of sessions	1 (9.09%)
**Content of the counseling sessions**	
Thought there was too much variety	1 (9.09%)
The variety was just right	10 (90.91%)
**Preferred modality of counseling sessions**	
Phone	3 (27.27%)
Zoom	8 (72.73%)
In-person	0 (0.00%)

Note. [a] $n = 1$ reported not relevant; [b] $n = 2$ reported not relevant; [c] $n = 1$ selected “other” and reported trouble accessing the app
