# Supplementary material for: Mindfulness-Based Ecological Momentary Intervention for Smoking Cessation to Address Cancer-Related Relapse Risk Factors: Intervention Development and Usability Findings
Source: Mindfulness (N Y). 2026 Mar 9;17(4):1101–19. doi: 10.1007/s12671-026-02775-0 (PMC12971066; doi:10.1007/s12671-026-02775-0)
Supplement: Supplementary file 5 — Supplementary file5 (PDF 267 kb) [file 12671_2026_2775_MOESM5_ESM.pdf]

Online Resource 5. Study 2: Treatment Feedback

Theme	Representative Quotes
**Usability of the app**	*"The app was pretty easy to use. There was really no effort involved with it. You just get a notification and then you go to the notification and then it either asks you to do the survey or the meditation, but yeah it was pretty simple to use."* (age 42, male, skin cancer)<br><br>*"I'm one of the people that are not tech savvy. I don't see anything that was difficult about downloading the app if you're interested in this type of program. It was pretty simple for me."* (age 60, female, breast cancer)<br><br>*"Having a smartphone is a little, tiny computer… having an app for it is really, really handy."* (age 61, female, breast cancer)
**App recommendations**	*"Say if it would come in at 9:00 am and then I wouldn't be able to look at until noon. So, for me it was just when the notifications would come up… or sometimes they would expire because I was too busy to look at them."* (age 42, male, skin cancer)<br><br>*"I think the reminders for the survey were infrequent enough. They were fine. For the rest of it, I really would've preferred being allowed to set up my own schedule…"* (age 68, female, gynecological cancer)
**Intervention content**	**Mindful Skills**<br><br>*"It just made me still for a while and relax and think of things, so I wasn't either up smoking or up screaming at somebody or just getting so mad, I was mad throughout the day. It helped me with reminding myself it's okay to be mad. It'll go away in a few minutes, and you can make the rest of the day better. It helped with that… Because smoking doesn't help you with that. Smoking just calms you down for a few more seconds. It comes back. You're so happy you're not thinking about it."* (age 66, female, thyroid cancer)<br><br>*"Some of the quotes were good. Some of them weren't relevant to me. I remember one was almost like a scare tactic, "You might get cancer one day." It's like, wow, I don't need to be reminded of that."* (age 42, male, skin cancer)<br><br>**Meditations**<br><br>*"I do long ones sometimes. They have some long ones that come up in the notification. That's the one I usually do sometimes in the morning. … I like both of them [short and long]. It's according to the way I'm feeling at that particular time. So, I choose which one I want to use. … I mean, my perspective is to help me lessen my smoking habits. That was one thing. To lessen my smoking habit. It also helps if I'm going through a stressful day. It's according to the way I'm feeling."* (age 60, female, breast cancer)<br><br>*"I guess I want to say a little frustrating. But again, it was not with the instructions, it was with the imposed timing [of the notifications]… I have to physically relocate myself to do those exercises because I don't want to be interrupted by the phone. … It would have been better for me to have self-scheduling or be encouraged to self-schedule."* (age 68, female, gynecological cancer)
**Overall feedback on the program**	**Acceptability**<br><br>*"Well, it was the kick in the butt that I needed. And I guess just the patches helped me and- well, and still the patches are helping. And the mindfulness, the exercises, kinda*

	of upped my – helped me be more aware of smoking- the habit of smoking. I can't really put it in words too good, but I'll try. Like instead of instinctively reaching for a cigarette, or in my case a cigar, I was able to stop and say, "Hey, slow down. Break the habit"." (age 51, male, bladder cancer)<br><br>**<u>Counseling</u>**<br><br>*"As I said, that [counseling] was one of my favorite features of the app. It helped me get sort of back on track because I was not on track. It added a personal component to the app that someone other than me cared about how I was progressing or what I might want to do to progress differently. So I really liked that contact."* (age 68, female, gynecological cancer)<br><br>**<u>Nicotine Patch</u>**<br><br>*"I tried the patches one time. Because I had melanoma before, the thought of putting chemicals on my skin again. It kinda scared me."* (age 42, male, skin cancer)
